# Regional Coleoptera Fauna: Applying Different Methods to Study Species Diversity in a Single Region

**DOI:** 10.3390/insects15120917

**Published:** 2024-11-24

**Authors:** Leonid V. Egorov, Sergei V. Dedyukhin, Sergei K. Alekseev, Olga S. Trushitsyna, Alexander B. Ruchin, Aleksey S. Sazhnev, Anna M. Nikolaeva, Mikhail N. Esin, Anatoliy A. Khapugin

**Affiliations:** 1 Prisursky State Nature Reserve, 428034 Cheboksary, Russia; platyscelis@mail.ru; 2 Joint Directorate of the Mordovia State Nature Reserve and National Park “Smolny”, 430005 Saransk, Russia; ded@udsu.ru (S.V.D.); esinmishka@gmail.com (M.N.E.); hapugin88@yandex.ru (A.A.K.); 3 Department of Botany, Zoology and Bioecology, Udmurt State University, 426034 Izhevsk, Russia; 4 Parks Directorate of Kaluga Region, 248000 Kaluga, Russia; stenus@yandex.ru; 5 Scientific Laboratory of Evolutionary Ecology, Ryazan State University, 390000 Ryazan, Russia; trushicina01@mail.ru; 6 Papanin Institute for Biology of Inland Waters, Russian Academy of Sciences, 152742 Yaroslavl, Russia; sazh@list.ru; 7 Oka State Nature Biosphere Reserve, 391072 Brykin Bor, Russia; nikolaeva.2005@mail.ru; 8 Institute of Environmental and Agricultural Biology (X-BIO), Tyumen State University, 625003 Tyumen, Russia

**Keywords:** pitfall traps, flight intercept traps, beer traps, Malaise traps, pan traps, Coleoptera, fauna, biodiversity

## Abstract

In order to study regional faunas, one or two methods of studying Coleoptera are often used. However, a comparison of several ways of studying shows that it is more expedient to use more than one of them. Six different methods of catching Coleoptera used in studies of the biodiversity of regional faunas were compared. This research employed pitfall traps (PfT), beer traps (BT), freely hanging flight intercept traps or window traps (FWT), Malaise traps (MT), pan traps (PT), and sweep net (SN). The goal was to assess the effectiveness of these methods in evaluating the biodiversity of this insect group. Studies were conducted from 2011 to 2017 and 2019 to 2023 in the Ryazan region (the central part of European Russia). A total of 27,892 specimens of Coleoptera (927 species from 64 families) were collected. Specifically, 17,659 specimens were collected using PfT (396 species from 36 families), 4971 specimens using BT (146 species from 30 families), 1014 specimens using FWT (221 species from 51 families), 109 specimens using MT (43 species from 16 families), 2220 specimens using PT (357 species from 42 families), and 1919 specimens using SN (265 species from 32 families). The maximum number of families was observed with FWT, while the minimum number of families was registered with MT. Interestingly, the highest number of rare species from both protected lists was obtained using BT and PfT. The Margalef index was higher for Coleoptera collections using PT and PfT, while the largest Shannon index values were recorded for samples using SN and PT. The Berger–Parker index was the highest for catches using BT, which was characterized by the highest dominance of one or more species. In our study, four species of Coleoptera were found that are included in the protected lists of the Russian Federation and 14 species of Coleoptera were listed in the protected species of the Ryazan region. Considering the results of this study, the Coleoptera fauna of the Ryazan region currently accounts for 1674 species.

## Simple Summary

We compare six different ways to explore Coleoptera: pitfall traps (PfTs), beer traps (BTs), freely hanging flight-intercept (window) traps (FWTs), Malaise traps (MTs), pan traps (PTs) and sweepnets (SNs). A total of 27,892 specimens of Coleoptera (927 species of 64 families) were collected. In total, 17,659 specimens were collected in PfTs (396 species), 4971 specimens with BTs (146 species), 1014 specimens with FWTs (221 species), 109 specimens in MTs (43 species), 2220 specimens in PTs (357 species) and 1919 specimens with the help of SNs (265 species). Interestingly, the highest number of rare species as indicated by protected lists were obtained using BTs and PfTs. The Margalef index was higher when collecting Coleoptera using PTs and PfTs. The highest values of the Shannon index were characterized by fees using SNs and PTs. The Berger–Parker index was the highest for catches in BTs. BT trapping was characterized by the highest values of dominance of one or more species. Taking the results of this study into account, the Coleoptera fauna of the Ryazan region now comprises 1674 species.

## 1. Introduction

Insects constitute the majority of both global and regional biodiversity in terms of species numbers [1]. They play a pivotal role in ecological processes and the functioning of all ecosystems. However, studying the structure of insect communities and local faunas is challenging due to difficulties in sampling and the need for taxonomic expertise across various insect families [2,3,4,5,6,7]. Among insects, Coleoptera exhibit particularly high species diversity in ecosystems, and our knowledge of their biodiversity is continuously expanding through the application of diverse research methodologies [8,9,10,11,12]. Despite this, Coleoptera faunas in many regions remain insufficiently studied, though these regional faunas often display patterns of significant ecological interest. These include variations in species richness, endemism, rarity, and community composition, all of which may exhibit distinct spatial and temporal patterns [4,13]. When the aim of a study is to create an inventory of a region’s fauna, it is essential to observe and document at least one specimen of each species present. Various collection techniques can be employed to achieve this. As such, substantial sampling effort is typically required to obtain accurate and reliable data on insect communities [2,14]. In practice, multiple collection methods should be used simultaneously to acquire a representative sample of regional Coleoptera faunas. However, logistical limitations often make this challenging for individual researchers [2,14,15,16,17].

For studies of regional faunas, one or two methods are often employed for sampling Coleoptera. Commonly used techniques include sweep nets and pitfall traps, while less frequently used methods include flight intercept traps, Malaise traps, pan traps, and beer traps [18,19,20,21,22]. However, comparative analyses of various sampling methods demonstrate that a comprehensive approach, involving multiple techniques, is more effective. For instance, Ong et al. [23] found that in the tropical forests of Borneo, baited pitfall traps and flight intercept traps captured complementary communities with distinct functional characteristics, suggesting that both should be used for assessing dung beetle biodiversity. In Michigan (USA), Haack and Ruesink [24] reported that 210 species were collected using baited multi-funnel traps, 104 species with pitfall traps, and 168 species with sweep nets. Similarly, in the beech forests of Golestan province (Iran), Rafiei-Jahed et al. [25] showed that window traps were more effective than other methods for sampling Coleoptera. Studies conducted in Norwegian forests revealed significant differences in the number of Coleoptera species and individuals captured by different trap types, with flight intercept and Malaise traps outperforming cross-pane traps [26]. Ranius and Jansson [27] demonstrated that using three methods—window traps, pitfall traps, and wood mould sampling—provided valuable data on Coleoptera fauna, as each method targeted different communities. Window traps, in particular, collected the highest number of species.

In this article, we aim to compare five types of traps commonly used in biodiversity research for the collection of Coleoptera. We will evaluate their effectiveness and capabilities in assessing Coleoptera biodiversity. The effectiveness of each trap is determined based on its ability to capture a higher number of Coleoptera species and individuals or a wider range of Coleoptera families. Therefore, the focus of this comparison will be on the trap’s performance in terms of the abundance and diversity of Coleoptera it captures, rather than from an economic or cost-efficiency perspective.

## 2. Materials and Methods

### 2.1. Study Area

The research was carried out from 2011 to 2017 and 2019 to 2023 in the Ryazan region, located in the center of European Russia. This region, covering an area of 39,600 km^2^, lies on the East European Plain, bordered by the Penza, Tambov, Lipetsk, Tula, Moscow, Vladimir, and Nizhny Novgorod regions, as well as the Republic of Mordovia. It is situated between latitudes 53° and 55° N and longitudes 55° and 59° E. The main part of the region falls within the Oka-Don and Meshchera Lowlands, while its southwestern area extends into the northeastern part of the Central Russian Upland.

**Figure 1:**
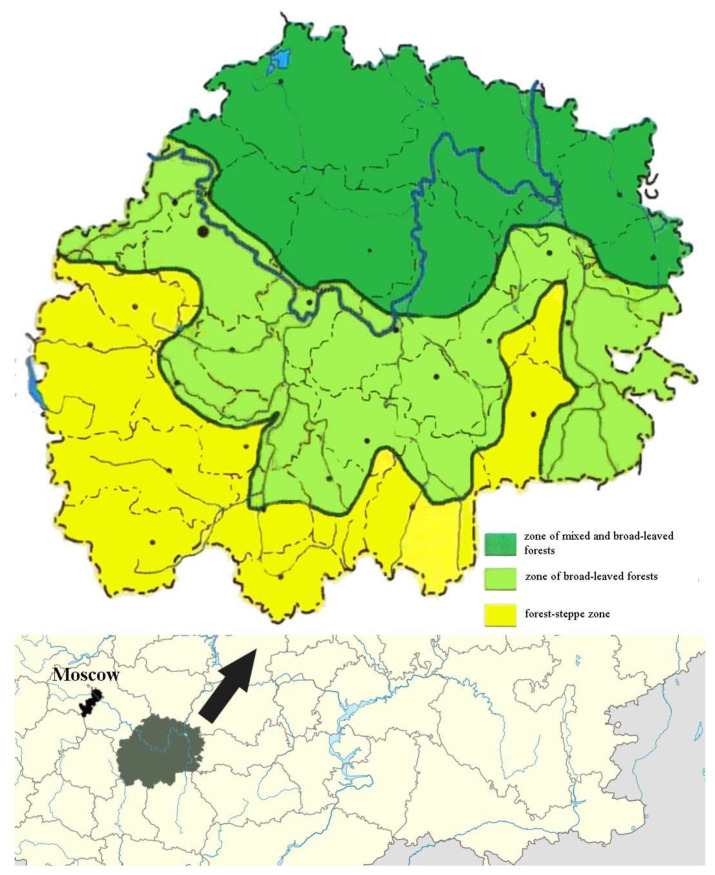
The location of the Ryazan region in the European part of Russia.

The climate of the Ryazan region is temperate continental, characterized by warm summers and moderately cold winters. Solar radiation across the region ranges from 90 to 95 kcal/cm^2^ per year, with annual precipitation varying from 700 mm in the northern part to 600 mm or less in the southwest. The northern areas, located on the left bank of the Oka and the right bank of the Moksha, experience excessive moisture, where precipitation exceeds evaporation [28]. Geographically, the region spans three distinct natural zones: the northern section belongs to the mixed coniferous–deciduous forest zone, the central area falls within the deciduous forest zone, and the southernmost parts extend into the forest–steppe zone (Figure 1). The river systems in the region are divided between the Caspian Sea basin (the Oka River and its tributaries) and the Sea of Azov basin (the Don River and its tributaries) [29,30].

### 2.2. Traps and Sampling Procedures

**Figure 2:**
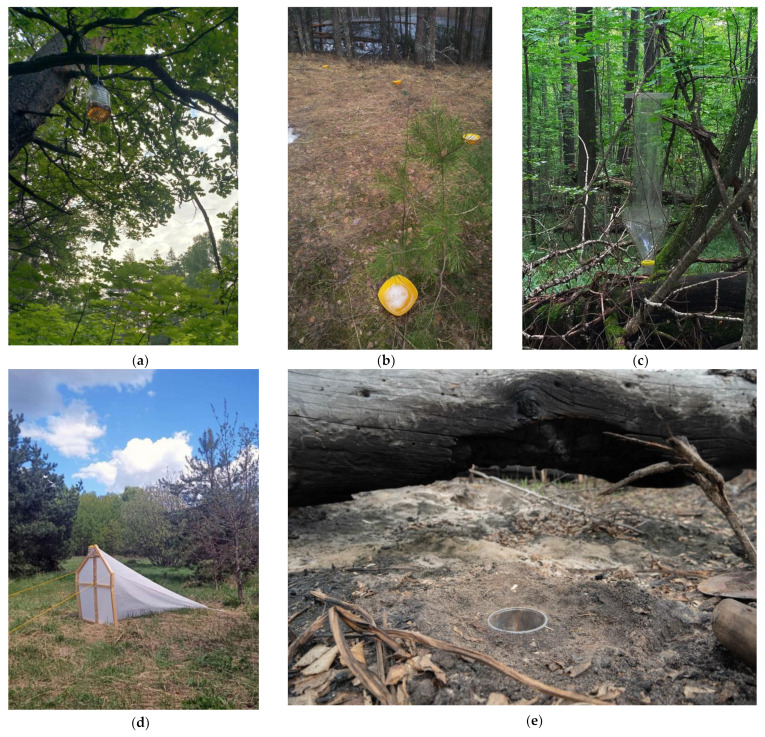
Various types of traps installed on the territory of the Ryazan region: (**a**) beer trap; (**b**) pan traps; (**c**) freely hanging flight intercept (window) trap; (**d**) Malaise trap; (**e**) pitfall trap.

Pitfall traps were deployed in a single biotope, arranged in a linear sequence (“trap line”) consisting of 10 traps positioned at intervals of 1.5 to 2 m. The traps consisted of plastic cups with an upper diameter of 93 mm, a lower diameter of 55 mm, and a height of 142 mm. A 4% formalin solution was used as a preservative. These traps were employed across both forested and open ecosystems, including meadows, steppes, clearings, and agroecosystems (Figure 2).

Beer traps consisted of 5 L plastic containers with a window cut 10 cm from the bottom on one side. These traps were suspended from tree branches at varying heights, ranging from 1.5 to 12 m above the ground [31]. As bait, fermenting beer with additives such as honey, jam, or sugar was used. Beer traps were exclusively deployed in forested ecosystems, including forest edges, along clearings, and within woodland interiors.

For freely hanging flight intercept (window) traps (FWT), transparent plastic traps based on designs from Cavaletto et al. [32] were used. One trap was set up at each sampling site. The collection containers beneath the traps were filled halfway with a 6% vinegar solution, which acted as a preservative, which was replaced during each trap check. The traps were suspended by ropes from tree branches. The trees were selected based on their location and suitability for bearing the trap’s weight, irrespective of species. The traps were placed at heights of 1.5 to 2 m from the ground, set both within forest interiors and along forest edges.

A homemade white trap, modeled after the Townes-style Malaise trap [33], was also used. The frame of the front screen was constructed using wooden uprights, while the trap itself was made of polyester. Collection tanks were filled with 70% ethanol as a preservative. For optimal efficiency, the traps were installed at forest edges or slightly within forest interiors, with the front screen oriented towards the light to attract flying insects.

For pan traps, we used yellow disposable plastic plates with a diameter of 21 cm and a capacity of 1.25 L. The plates were filled to two-thirds with water mixed with detergent to break the surface tension. Between 7 and 10 traps were arranged in a linear position on the ground surface (either within grass or directly on the soil), with 3 m intervals between traps. Sample collection occurred every 3 to 7 days, depending on the accessibility of the site. Pan traps were employed in both forested and open habitats.

Sweep nets were employed to sample terrestrial Coleoptera from herbaceous vegetation and low-growing shrubs. A standard entomological net was used for this method. This approach also included examining flowering plants and capturing anthophilous (flower-associated) Coleoptera using an aerial net. Additionally, sweep nets involved searching for beetles under rocks and on the ground.

### 2.3. Data Analyses

The nomenclature of Coleoptera was verified and updated in accordance with the latest catalogs [34,35,36,37,38,39,40] and other contemporary publications [41,42]. Species lists within families were revised using recent data on individual taxonomic groups [43,44,45]. The years of species descriptions for certain Coleoptera were specified based on Bousquet [46]. The identification of the samples was carried out by S. Alekseev and O. Trushitsyna (Carabidae), S. Kurbatov and O. Semionenkov (Staphylinidae), and S. Tshernyshev (some Byrrhidae). All other samples from other families, as well as a complete analysis of the list of species, were supervised by L. Egorov.

To compare the Coleoptera fauna captured by different trap types, we employed the Jaccard index, which assesses the similarity between faunal compositions. To analyze species diversity, dominance, and the evenness of faunal distribution, we calculated several biodiversity metrics, including the Margalef index, the Berger–Parker index, the Shannon index, and the Simpson index [47,48]. The non-parametric Kruskal–Wallis test (*p* < 0.05) was used to test the difference in the means of coleopterans caught by different collection methods. Differences between the number of caught specimens and species using different methods were estimated using the non-parametric Mann–Whitney test. Statistical treatments and visualization were performed using PAST software [49]. Data for the number of species and individuals recorded during the study period were pooled to obtain individual based rarefaction curves at 95% confidence levels to determine the sampling effort of various traps using the PAST software version 4.11 [49].

## 3. Results

**Figure 3:**
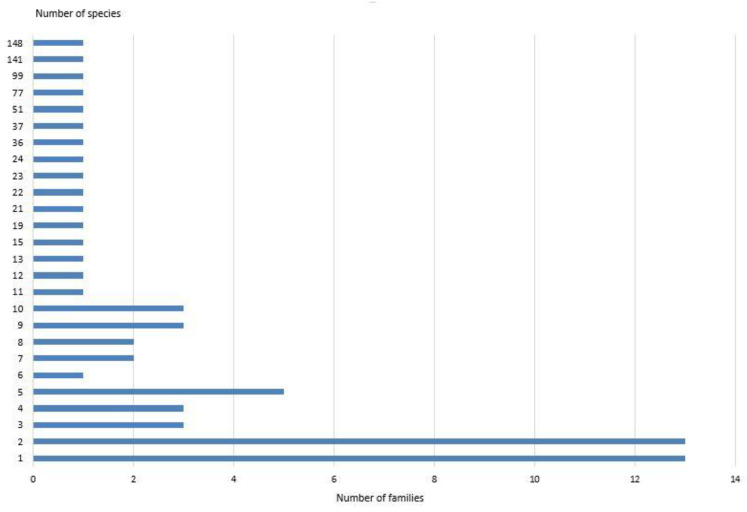
Distribution of Coleoptera families by the number of species in total by all collection methods.

During this research, we recorded data on 927 species of Coleoptera from 64 families (Table 1). The families with the highest species richness were Carabidae (148 species), Curculionidae (141 species), and Chrysomelidae (99 species) (Appendix A Table A1 and Figure 3). Thirteen families were represented in our study by only one species (Hydraenidae, Eucinetidae, Lampyridae, Lymexylidae, Byturidae, Trogossitidae, Sphindidae, Cryptophagidae, Silvanidae, Cucujidae, Laemophloeidae, Anamorphidae, and Orsodacnidae) or two species (Trogidae, Lucanidae, Geotrupidae, Heteroceridae, Throscidae, Lycidae, Monotomidae, Kateretidae, Cerylonidae, Ciidae, Pyrochroidae, and Salpingidae).

Of the 927 identified species, 323 species (34.8%) were represented by only one specimen across all collection methods, while 112 species (12.1%) were represented by two specimens and 62 species (6.7%) by three specimens (Appendix A Table A1). Notably, ten species (*Harpalus rufipes* (De Geer, 1774), *Pterostichus quadrifoveolatus* (Letzner, 1852), *Hylobius abietis* (Linnaeus, 1758), *Strophosoma capitatum* (De Geer, 1775), *Carabus arvensis* (Herbst, 1784), *Cryptarcha strigata* (Fabricius, 1787), *Pterostichus oblongopunctatus* (Fabricius, 1787), *Soronia grisea* (Linnaeus, 1758), *Pterostichus melanarius* (Illiger, 1798), and *Dermestes laniarius* Illiger, 1801) accounted for 44.7% of all identified specimens (Appendix A Table A1).

In our study, representatives from only ten Coleoptera families were collected using all employed trapping methods. These families included Carabidae, Scarabaeidae, Elateridae, Cantharidae, Dermestidae, Coccinellidae, Cerambycidae, Chrysomelidae, Brentidae, and Curculionidae. Notably, only two species, *Agrypnus murinus* (Linnaeus, 1758) and *Strophosoma capitatum*, were captured by all collection methods (Table 1 and Appendix A Table A1).

The total number of both species (χ^2^ = 35.66; *p* < 0.0001) and individuals (χ^2^ = 34.91; *p* < 0.0001) differed significantly between the collection methods used in this study. This indicates the significant differences between the efficiency of different collection methods for the investigation of Coleoptera fauna.

The pairwise Mann–Whitney test demonstrated that the number of species and specimens collected using Malaise traps was significantly lower (*p* < 0.001) compared to all other trapping methods. Furthermore, the data indicated that the catch of Coleoptera species and specimens in beer traps was significantly less than that obtained from freely hanging flight intercept traps (*p* < 0.001) and pan traps (*p* < 0.05). However, the number of species collected by beer traps did not significantly differ from that obtained using sweep nets (*p* > 0.05).

The capture of different Coleoptera families and species varied significantly among the trapping methods employed (Table 1 and Table 2). Pitfall traps and pan traps yielded the highest species diversity, while Malaise traps resulted in the lowest number of captured species. This trend was also reflected in the number of species unique to each trapping method, with Malaise traps showing a similar pattern.

In terms of family diversity, the freely hanging flight intercept traps recorded the maximum number of families, with 51, whereas the Malaise traps registered the minimum. The total number of specimens collected through various trapping methods also exhibited significant differences. The highest number of Coleoptera specimens was obtained from pitfall traps, while the lowest was recorded from Malaise traps.

Interestingly, despite the lower overall species diversity (only 221 species) associated with freely hanging flight intercept traps, this method captured the largest proportion of families that were not represented by other trapping methods, accounting for 8 families or 15.7% of the total.

In our research, we identified 14 Coleoptera species that are included in the protected species lists of the Ryazan region [50], along with 4 species listed in the national protected species lists of Russia [51]. Notably, the maximum number of rare species from both protected lists was captured using pitfall traps and beer traps. In contrast, no protected species were recorded from Malaise traps or pan traps (Table 2).

Considering the findings of this study alongside previously published information [52,53,54,55,56,57], the total known Coleoptera fauna of the Ryazan region now accounts for 1674 species.

The largest number of newly recorded species for the region has been found among the multi-species families Chrysomelidae and Curculionidae. Conversely, only five species were identified within the family Carabidae (ground beetles), which can be attributed to extensive prior studies on this group in the Ryazan region [58]. The most significant contributions to the regional fauna came from freely hanging flight intercept traps (34.4%) and pan traps (29.4%) (Table 2).

**Figure 4:**
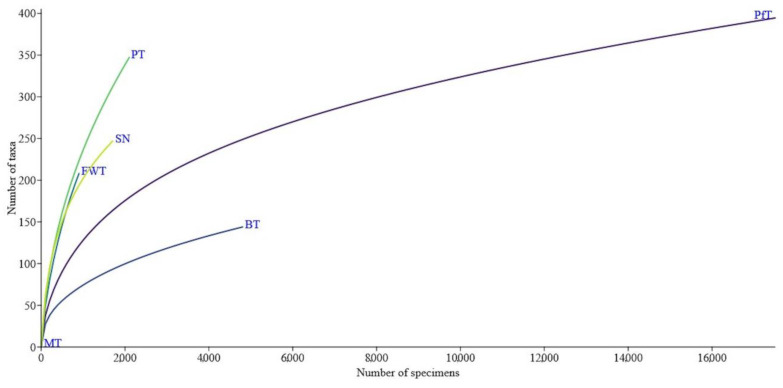
Differences in species richness of beetles in different trapping methods. Note: pitfall traps (PfT), beer traps (BT), freely hanging flight intercept traps or window traps (FWT), Malaise traps (MT), pan traps (PT), and sweep net (SN).

The species richness was significant when using pitfall traps (Figure 4). At the same time, a small number of individuals and species using other methods did not give a similar result. It can be predicted that the use of other methods, with the exception of beer traps, will bring even greater species richness. It is especially necessary to highlight pan traps, which, with a small number of specimens in traps, gave a sufficiently high biodiversity. Let us assume that the use of pan traps in a wide variety of biotopes at different times of the year can have a greater effect than the use of pitfall traps. At the same time, the use of even more beer traps would be unlikely to become effective.

**Figure 5:**
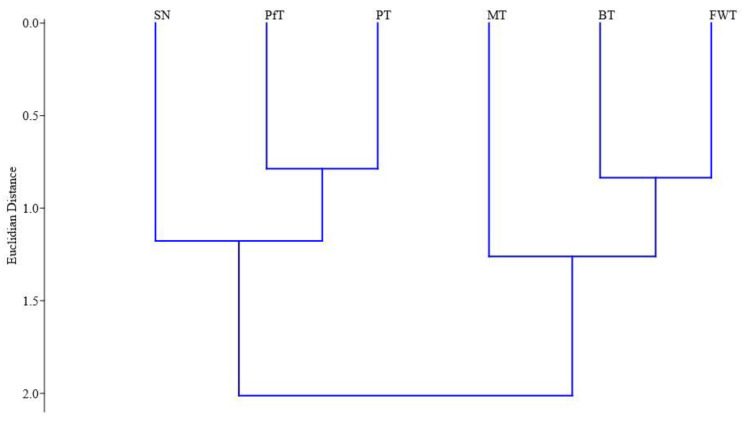
The similarity of Coleoptera species in different trapping methods based on the Jacquard index (Euclidean distance). Note: pitfall traps (PfT), beer traps (BT), freely hanging flight intercept traps or window traps (FWT), Malaise traps (MT), pan traps (PT), and sweep net (SN).

The Margalef index exhibited higher values for pan traps and pitfall traps (Table 1). In contrast, the lowest values of this index were recorded from Malaise traps and beer traps. The Shannon index demonstrated the highest values in collections made using entomological nets and pan traps, while the lowest values were observed in samples collected with beer traps. The Berger–Parker index was highest for specimens collected from beer traps, indicating a greater dominance of certain species. Conversely, the lowest value of this index was recorded from the collections made with sweep nets. A similar trend was noted with the Simpson index, where beer traps were characterized by a pronounced dominance of one or more species. Specifically, over half of all individuals (55.5%) captured in beer traps belonged to the families Nitidulidae (notably, *Cryptarcha strigata* (Fabricius, 1787), *Soronia grisea* (Linnaeus, 1758), and *Glischrochilus grandis* (Tournier, 1872)) and Scarabaeidae (*Protaetia marmorata* (Fabricius, 1792)), which significantly influenced the results. The calculation of the Jaccard index revealed distinct differences among the trapping methods (Figure 5). Two methods, pan traps and pitfall traps, clustered together, indicating similarities in biodiversity. The biodiversity obtained with the sweep net was also found to be closely aligned with this cluster. In contrast, beer traps and freely hanging flight intercept traps formed a separate group. Malaise traps were positioned near this cluster, indicating some degree of similarity in the captured fauna.

## 4. Discussion

The primary methods for studying regional insect fauna include sampling techniques such as sweep nets, pitfall traps, and light traps [59,60,61]. Less frequently employed methods include window traps, pan traps, and Malaise traps [62,63,64,65]. In recent years, the use of traps with various pheromones and fermenting baits has increased significantly [11,12,18,66].

Different trapping methods are often utilized based on the specific taxonomic or ecological groups of insects being studied. For instance, pan traps and Malaise traps are commonly employed for sampling Hymenoptera [63,64]. In contrast, collections of Coleoptera are typically conducted using a range of differentiated methods tailored to various environmental contexts. Pitfall traps are primarily used to sample soil-dwelling beetles, while flight intercept traps, light traps, Malaise traps, and beer traps are utilized for capturing flying forms [12,65,67].

However, individual sampling methods may not always provide a comprehensive assessment of the Coleoptera fauna within a specific region, often referred to as the regional fauna. This limitation is particularly relevant for studying phytophagous, saproxylic, and rare species. Moreover, the upper strata of forests remain insufficiently explored, despite the potential for high biodiversity in these areas.

Pitfall traps are the most commonly employed method in Coleoptera research due to their ease of use and cost-effectiveness compared to many other sampling techniques [2]. These traps are particularly effective for studying well-defined families, as they primarily collect species that inhabit litter and the soil surface. This method facilitates the simultaneous collection of a large number of samples from multiple locations, which is essential for subsequent statistical analyses.

However, there are limitations associated with the use of pitfall traps. These limitations primarily pertain to the interpretation of results and the method’s focus on a narrow range of ecological groups within the Coleoptera [68,69]. In our studies, pitfall traps yielded the highest number of species and specimens collected; however, they ranked third in terms of family diversity. Notably, despite the significant volume of samples obtained, no rare species listed on the federal protected list were captured using this method. In summary, while pitfall traps provide a substantial volume of data and are convenient for studying epigeal fauna, their limitations in capturing certain ecological groups and rare species should be acknowledged.

Beer traps have gained significant popularity in recent years as an effective method for collecting Coleoptera. These traps utilize beer-based baits, often enhanced with sugar-containing liquids and, occasionally, fruits to attract various insect species [11,18,31,66,70]. The primary method of deployment involves suspending the traps from tree branches at varying heights. However, practical applications have demonstrated that beer traps can also be successfully employed in open biotopes when mounted on specialized devices [67]. The Coleoptera predominantly captured in these traps include saproxylous species, anthophilous insects, and those that feed on tree sap [18,30,71]. It is noteworthy that the Coleoptera fauna collected from beer traps often differs from those obtained through other sampling methods. This variation likely accounts for the substantial number of rare species within the regional fauna that have been included in protected lists, primarily due to the limited data available from studies conducted using other methods.

Freely hanging flight intercept traps are primarily effective at collecting flying individuals; however, they typically do not capture the majority of flightless species that inhabit the litter layer [2,72]. These traps have demonstrated their utility in studying rare Coleoptera species and are frequently employed in the investigation of saproxylous species [73,74,75,76]. Similar to beer traps, flight intercept traps are often suspended from tree branches, and their placement near trunks can result in the capture of species that traverse along the trunks and branches. In our study, the highest number of families was recorded using this trap type compared to other methods, a finding consistent with surveys conducted in forest sites in Hong Kong [77].

In contrast, Malaise traps are infrequently utilized in Coleoptera studies due to their limited effectiveness in capturing a diverse range of species and families [26,72,77,78]. However, this type of trap is stationary and can yield favorable results as an auxiliary trapping method throughout the season [65,79]. Various factors influence the number of individuals and species captured, with the trap’s location in the biotope, its design, and the size of the collection cells being critical determinants [79].

Pan traps are a passive sampling method that does not require bulky or specialized equipment, making them an effective tool for assessing the diversity of flying insect pollinators [80,81]. Typically, these traps consist of plastic containers or plates in various colors, which attract different groups of insects based on their visual perception and color preferences associated with flowers [82].

Although the researcher does not directly influence the catches, several factors can affect the quality and quantity of insects trapped. First, color preferences may significantly impact overall catch rates, as insects that visit flowers often show a preference for specific colors. Additionally, smaller individuals and species are more likely to enter pan traps, as they can avoid larger traps more easily. Other important factors include the size of the traps, their quantity, the location within the biotope, the surrounding vegetation, and the visibility range of the traps [19,81,83,84,85,86]. In our research, the use of pan traps yielded valuable insights into the overall biodiversity of Coleoptera; however, they were less effective for studying rare species. The diversity metrics at both the family and species levels ranked second in our findings. Notably, a significant percentage of species was discovered exclusively through this method.

Insect netting is generally conducted in two primary ways: targeted trapping using an aerial net and collecting insects from vegetation with a sweep net. Additionally, Coleoptera can be collected through visual inspection of flowering plants, branches, and leaves; under stones; on stumps; and of other surfaces [24,87,88]. This method is often a form of selective collection that depends on the researcher’s knowledge of species biology, the timing and location of collection, the specific biotope, and other environmental factors [24,89,90]. In our research, using sweep nets allowed us to identify a significant percentage of species and families not captured by other methods. However, it is worth noting that rare species were often underrepresented in this collection method.

The Margalef index measures species richness in a specific area, with higher values indicating greater species diversity [91,92]. In our study, the greatest species richness was achieved using two methods: pan traps and pitfall traps. Pan traps are commonly employed to capture flying insects attracted to various flower-like colors that mimic blooming plants. Hymenoptera and Diptera are frequently caught in these traps, but various families of Coleoptera can also be represented [80,81,93,94]. On the other hand, pitfall traps offer a simple and cost-effective method for sampling terrestrial arthropods and have been used extensively in studies examining the abundance and diversity of families such as Carabidae and Staphylinidae [95,96,97,98]. These families formed the core of the studied fauna collected from these traps. The very low Margalef index value observed when using Malaise traps highlights the limited applicability of this method for studying Coleoptera. Previously, statistical analyses were performed on Diptera, Hymenoptera, and Lepidoptera only, as beetles were insufficiently represented [65,79,94]. In broader studies, investigations have shown that Coleoptera constitute less than 2% of the total insect population captured by Malaise traps [99]. Despite this, smaller traps are often utilized for analyzing Coleoptera fauna, particularly in studies involving molecular analysis due to their ability to collect material without attractants [84,100,101].

The Shannon index characterizes diversity and evenness within a community, typically reflecting high diversity and strong community structure [92,102]. Our findings indicate that pan traps and entomological nets recorded a more diverse Coleoptera fauna. The Berger–Parker index expresses the relative abundance of the most common species, with an increase in this index signifying both increased diversity and the greater dominance of specific species. Similar trends were observed with the Simpson index [91,102].

Notably, several species and families dominated in catches from beer traps, primarily represented by the Nitidulidae family [103,104,105,106]. This method can also capture significant representatives like *Cryptarcha strigata*, *Soronia grisea*, and *Glischrochilus grandis* [12,107,108]. Therefore, in certain cases, it may be advisable to conduct statistical analyses excluding representatives of the Nitidulidae family to ensure more accurate results [106].

When comparing biodiversity captured using different trapping methods, we identified two distinct clusters. The first cluster comprised pan traps, pitfall traps, and sweep nets, all of which were closely related in terms of the types of Coleoptera they captured. Pan traps and pitfall traps are primarily designed to capture terrestrial beetles or those flying near grass cover [2,60,96,109]. A similar relationship was observed in another study [97].

Pitfall traps are placed in the ground, while pan traps are positioned on the soil surface among grasses. Sweep netting, along with visual inspections, typically focuses on Coleoptera found on the soil surface, herbaceous plants, and flowering plants [110]. In these methods, researchers can effectively capture beetles that are within arm’s reach using an entomological net [109,111]. Thus, the grouping of these three methods into a single large cluster is logical and reflects their similar ecological focus.

On the other hand, the second large cluster included beer traps and freely hanging flight intercept traps, with Malaise traps showing similar biodiversity positioned nearby. The first two types of traps are typically used to capture actively flying insects in the shrub layer and understory. A distinguishing feature is that beer traps use beer as bait, while freely hanging flight intercept traps do not rely on bait [2,105,106,112]. Despite this difference, both trap types captured a similar faunal composition. This similarity explains the recommendation to use baited traps in conjunction with freely hanging flight intercept traps as complementary methods [75]. The placement of these traps at a specific height above the ground likely contributes to the similarity in Coleoptera fauna captured. Additionally, Malaise traps, classified as aerial traps [97], were included in this cluster, although they primarily captured a limited number of species, mostly phytophagous beetles, as they were also set up at ground level during our studies.

## 5. Conclusions

Various trapping methods, when applied correctly, can effectively monitor biodiversity and be used to study rare insect species that are difficult to detect through other means. When compiling lists of regional fauna species, it is crucial to understand the impact of different sampling methods on the results. Utilizing more than one method is especially important, as most on-site sampling regimes are often not intensive enough to thoroughly study any ecological or taxonomic group of Coleoptera in a given region. Selecting an appropriate collection technique for Coleoptera is vital for accurately assessing the biodiversity of the regional fauna. Our study confirmed the significance of the method used for studying Coleoptera in assessing faunal diversity. Over several years, we collected 27,892 specimens, representing 927 species from 64 families. The largest number of species belonged to the families Carabidae, Curculionidae, and Chrysomelidae. The method of sample collection significantly influenced the contribution of each technique to the overall results. The greatest species diversity was obtained from pitfall traps and pan traps, while the fewest species were captured in Malaise traps. Notably, the maximum number of families was recorded using freely hanging flight intercept traps, while the minimum was observed in Malaise traps. In our research, we identified four Coleoptera species listed as protected by the Russian Federation, along with 14 species included in the protected lists for the Ryazan region. Interestingly, the highest numbers of rare species from both protected lists were obtained using beer traps and pitfall traps. To effectively study the regional fauna of Coleoptera, we recommend employing at least four collection methods: pitfall traps, beer traps, pan traps, and freely hanging flight intercept traps. These methods cover complementary communities, including soil fauna, herbaceous and shrubby tiers, and the canopy of trees. In addition, it should be taken into account that our results were obtained on a limited scale in a temperate climate zone. It is possible that in other ecosystems and using other research methods, the results may differ from our results.

## Figures and Tables

**Table 1:** The number of species (above) and specimens (below) in families falling into different types of traps.

Family	PfT	BT	FWT	MT	PT	SN	Total
Carabidae	136 10,821	1 1	3 8	1 1	45 142	13 33	148 11,006
Dytiscidae	5 6		2 4		7 8	2 8	13 26
Hydrophilidae	5 5		4 5		7 25	2 4	12 39
Histeridae	4 23	1 1	2 5		4 4	10 33	10 33
Hydraenidae			1 1				1 1
Leiodidae	5 10	1 1	10 15		7 15		19 41
Staphylinidae	53 930	10 76	21 131		13 45	10 20	77 1202
Trogidae	2 39						2 39
Lucanidae	2 36	1 1	2 7		1 1		2 45
Geotrupidae	1 149		1 26		1 2	1 5	2 182
Scarabaeidae	7 77	7 936	8 52	1 1	13 35	15 174	22 1275
Scirtidae		1 2	6 8	1 2		7 23	10 35
Eucinetidae	1 1		1 1		1 2		1 4
Buprestidae			2 4		5 19	4 34	10 57
Byrrhidae	8 146				1 1		9 147
Heteroceridae					2 4		2 4
Eucnemidae	1 1		4 9				5 10
Throscidae	1 17		2 30		1 1	1 1	2 49
Elateridae	11 354	16 256	20 209	5 6	12 154	13 73	36 1052
Lycidae	2 2		1 2			1 58	2 62
Lampyridae	1 5		1 7				1 12
Cantharidae	4 9	8 207	3 7	6 30	10 51	13 100	21 404
Dermestidae	2 651	5 30	1 2	1 1	1 41	2 18	9 743
Ptinidae	1 9	4 4	11 20		2 2		11 35
Lymexylidae			1 1				1 1
Byturidae					1 19	1 7	1 26
Trogossitidae			1 1				1 1
Cleridae	1 1	1 6	3 3			2 45	5 55
Melyridae		3 7	3 16	3 9	7 64	6 136	8 232
Sphindidae	1 1						1 1
Erotylidae	3 17	1 1	5 13		1 6	1 1	7 38
Monotomidae		1 6	1 1		1 1		2 8
Kateretidae					1 1	1 3	2 4
Nitidulidae	2 2	14 2643	11 68		1 1	2 2	23 2716
Cryptophagidae						1 1	1 1
Silvanidae		1 1					1 1
Cucujidae			1 18				1 18
Phalacridae						4 46	4 46
Laemophloeidae			1 1				1 1
Cerylonidae			2 2				2 2
Latridiidae	1 1		5 16		2 11		6 28
Anamorphidae			1 1				1 1
Endomychidae	2 8		1 3		1 1		3 12
Coccinellidae	7 11	5 6	5 5	9 23	21 142	19 174	37 361
Mycetophagidae		1 6	5 23				5 29
Ciidae			2 2				2 2
Melandryidae		2 2	4 10		1 1		7 13
Zopheridae			1 1		2 2		3 3
Mordellidae	1 1	1 1	2 2		1 1	6 42	8 47
Tenebrionidae	11 943		5 20	1 6	5 93	3 81	15 1143
Oedemeridae		2 4	3 5		6 45	9 132	9 186
Meloidae	5 25				1 19		5 44
Pyrochroidae		2 9	1 2	1 1			2 12
Salpingidae			2 3				2 3
Anthicidae	1 1				2 8	2 3	3 12
Aderidae					2 13		2 13
Scraptiidae		1 1	1 2		1 1	2 4	4 8
Cerambycidae	11 64	25 626	18 32	1 1	9 12	22 270	51 1005
Orsodacnidae		1 1		1 2			1 3
Chrysomelidae	27 69	12 70	10 14	6 8	58 724	49 192	99 1077
Anthribidae	1 10	2 4	3 5	1 1	1 1		5 21
Attelabidae			1 1		4 4		4 5
Brentidae	9 10	2 3	1 1	1 1	15 34	7 17	24 66
Curculionidae	61 3204	14 59	15 189	4 16	80 464	34 179	141 4111
Total number of families	36	30	51	16	42	32	64
Total number of species	396	146	221	43	357	265	927
Total number of specimens	17,659	4971	1014	109	2220	1919	27,892

Note: pitfall traps (PfT), beer traps (BT), freely hanging flight intercept traps or window traps (FWT), Malaise traps (MT), pan traps (PT), and sweep net (SN).

**Table 2:** The total indicators for the use of different methods of catching Coleoptera obtained in studies in the Ryazan region.

Index	PfT	BT	FWT	MT	PT	SN	Total
The number of families caught only by this collection method	2	1	8	0	1	2	-
The proportion of families caught only by this collection method from the total number of families caught by this collection method	5.6	3.3	15.7	0	2.4	6.3	-
The number of species caught only by this collection method	213	44	102	5	146	91	-
The proportion of species caught only by this collection method from the total number of species caught by this collection method	53.8	30.1	46.2	11.6	40.9	34.3	-
The number of species that are new for the fauna of the region	85	30	76	8	105	49	285
The proportion of species that are new for fauna of the region from the total number of species caught by this collection method	21.5	20.5	34.4	18.6	29.4	18.5	30.7
The number of species that are new for fauna of the region caught only by this collection method	62	13	58	4	72	28	-
The proportion of species that are new for fauna of the region caught only by this collection method from the total number of species caught by this collection method	72.9	43.3	76.3	50.0	68.6	57.1	-
Margalef index	40.4	17.0	31.9	8.95	46.2	34.8	-
Berger–Parker index	0.11	0.24	0.14	0.16	0.17	0.04	-
Shannon index	3.74	3.00	4.15	3.27	4.52	4.74	-
Simpson index	0.05	0.11	0.04	0.05	0.04	0.01	-
The number of species included in the Red Data Book of the Russian Federation	0	3	1	0	0	1	4
The number of species included in the Red Data Book of the Ryazan region	7	7	1	0	0	2	14

Note: pitfall traps (PfT), beer traps (BT), freely hanging flight intercept traps or window traps (FWT), Malaise traps (MT), pan traps (PT), and sweep net (SN).

## Data Availability

The data presented in this study are available in the article.

## Appendix A

**Table A1:** Number and biodiversity of Coleoptera collected using various methods.

Family, Species	PfT	BT	FWT	MT	PT	SN	Total
**Carabidae**							
*Acupalpus exiguus* (Dejean, 1829)					1		1
*Acupalpus meridianus* (Linnaeus, 1761)					2		2
*Agonum fuliginosum* (Panzer, 1809)	4						4
*Agonum gracile* (Sturm, 1824)	1						1
*Agonum gracilipes* (Duftschmid, 1812)	56						56
*Agonum lugens* (Duftschmid, 1812)	2				1		3
*Agonum sexpunctatum* (Linnaeus, 1758)	9						9
*Agonum viduum* (Panzer, 1796)	3						3
*Agonum versutum* (Sturm, 1824)	2				1		3
*Amara aenea* (De Geer, 1774)	37				1		38
*Amara apricaria* (Paykull, 1790)	1						1
*Amara aulica* (Panzer, 1796)	23				17	2	42
*Amara bifrons* (Gyllenhal, 1810)	53				1		54
*Amara brunnea* (Gyllenhal, 1810)	2						2
*Amara communis* (Panzer, 1797)	120				2		122
*Amara consularis* (Duftschmid, 1812)	9				1		10
*Amara convexior* (Stephens, 1828)	9				1		10
*Amara eurynota* (Panzer, 1796)	12						12
*Amara famelica* (C.C.A. Zimmermann, 1832)	1						1
*Amara familiaris* (Duftschmid, 1812)	9				4		13
*Amara ingenua* (Duftschmid, 1812)	1						1
*Amara lunicollis* (Schiødte, 1837)	7						7
*Amara montivaga* (Sturm, 1825)	1						1
*Amara nitida* (Sturm, 1825)	1						1
*Amara ovata* (Fabricius, 1792)	4						4
*Amara plebeja* (Gyllenhal, 1810)					2	1	3
*Amara praetermissa* (C.R. Sahlberg, 1827)	4						4
*Amara similata* (Gyllenhal, 1810)	12						12
*Amara tibialis* (Paykull, 1798)	11				2		13
*Anchomenus dorsalis* (Pontoppidan, 1763)	1						1
*Anisodactylus binotatus* (Fabricius, 1787)	3						3
*Anisodactylus nemorivagus* (Duftschmid, 1812)	3						3
*Anisodactylus signatus* (Panzer, 1796)	5						5
*Badister bullatus* (Schrank, 1798)	19						19
*Badister dilatatus* (Chaudoir, 1837)	1		1			1	3
*Badister lacertosus* (Sturm, 1815)	23						23
*Badister sodalis* (Duftschmid, 1812)	2						2
*Badister unipustulatus* (Bonelli, 1813)					1		1
*Bembidion azurescens* (Dalla Torre, 1877)						2	2
*Bembidion dentellum* (Thunberg, 1787)	3						3
*Bembidion gilvipes* (Sturm, 1825)	3				1		4
*Bembidion guttula* (Fabricius, 1792)	1						1
*Bembidion lampros* (Herbst, 1784)	17						17
*Bembidion litorale* (G.-A. Olivier, 1790)						5	5
*Bembidion properans* (Stephens, 1828)	2						2
*Bembidion ruficolle* (Panzer, 1796)						6	6
*Bembidion quadrimaculatum* (Linnaeus, 1761)	10						10
*Bembidion schueppelii* Dejean, 1831)	1						1
*Bembidion striatum* (Fabricius, 1792)						7	7
*Bembidion velox* (Linnaeus, 1761)						4	4
*Broscus cephalotes* (Linnaeus, 1758)	1						1
*Calathus erratus* (C.R. Sahlberg, 1827)	25				2		27
*Calathus fuscipes* (Goeze, 1777)	5				3		8
*Calathus melanocephalus* (Linnaeus, 1758)	12				23		35
*Calathus micropterus* (Duftschmid, 1812)	350		6				356
*Callistus lunatus* (Fabricius, 1775) **	2						2
*Calosoma inquisitor* (Linnaeus, 1758) **	10						10
*Calosoma investigator* (Illiger, 1798) **	1						1
! *Calosoma maderae* (Fabricius, 1775)	2						2
*Carabus arvensis* (Herbst, 1784)	1268					1	1269
*Carabus cancellatus* (Illiger, 1798)	495				1		496
*Carabus clathratus* (Linnaeus, 1761) **	6						6
*Carabus convexus* (Fabricius, 1775)	12						12
*Carabus glabratus* (Paykull, 1790)	74		1				75
*Carabus granulatus* (Linnaeus, 1758)	243				1		244
*Carabus hortensis* (Linnaeus, 1758)	95						95
*Carabus marginalis* (Fabricius, 1794) **	1						1
*Carabus nemoralis* (O.F. Müller, 1764)	21						21
*Carabus violaceus aurolimbatus* (Dejean, 1830) **	4						4
*Chlaenius nigricornis* (Fabricius, 1787)	4						4
*Cicindela campestris* (Linnaeus, 1758)	10						10
*Cicindela hybrida* (Linnaeus, 1758)	13						13
*Cicindela sylvatica* (Linnaeus, 1758)	1						1
*Clivina fossor* (Linnaeus, 1758)	1						1
*Cychrus caraboides* (Linnaeus, 1758)	11						11
*Cymindis angularis* (Gyllenhal, 1810)	2						2
*Dolichus halensis* (Schaller, 1783)	8						8
! *Drypta dentata* (P. Rossi, 1790)	2				1		3
*Dyschirius globosus* (Herbst, 1784)	1						1
*Dyschirius tristis* (Stephens, 1827)	1						1
*Elaphrus cupreus* Duftschmid, 1812)	4						4
*Elaphrus riparius* (Linnaeus, 1758)	1						1
*Harpalus affinis* (Schrank, 1781)	29				1		30
*Harpalus anxius* (Duftschmid, 1812)	92					1	93
*Harpalus calceatus* (Duftschmid, 1812)	4						4
*Harpalus distinguendus* (Duftschmid, 1812)	50						50
*Harpalus griseus* (Panzer, 1796)	59			1	2		62
*Harpalus hirtipes* (Panzer, 1796)	2						2
*Harpalus laevipes* (Zetterstedt, 1828)	13						13
*Harpalus latus* (Linnaeus, 1758)	25				2		27
*Harpalus luteicornis* (Duftschmid, 1812)	3				3		6
*Harpalus picipennis* (Duftschmid, 1812)					1		1
*Harpalus progrediens* (Schauberger, 1922)	18						18
*Harpalus pumilus* (Sturm, 1818)	14						14
*Harpalus rubripes* (Duftschmid, 1812)	187				2		189
*Harpalus rufipes* (De Geer, 1774)	1936				13		1949
*Harpalus signaticornis* (Duftschmid, 1812)	25				3		28
*Harpalus smaragdinus* (Duftschmid, 1812)	18						18
*Harpalus tardus* (Panzer, 1796)	26				3		29
*Harpalus xanthopus winkleri* (Schauberger, 1923)	35						35
*Lebia chlorocephala* (J.J. Hoffmann, 1803)	1						1
*Lebia cruxminor* (Linnaeus, 1758)	2				1		3
*Leistus ferrugineus* (Linnaeus, 1758)	11				10		21
*Leistus terminatus* (Panzer, 1793)	4				3		7
*Licinus depressus* (Paykull, 1790)	5						5
*Limodromus assimilis* (Paykull, 1790)	406	1					407
*Limodromus krynickii* (Sperk, 1835)	10						10
*Loricera pilicornis* (Fabricius, 1775)	4						4
*Masoreus wetterhallii* (Gyllenhal, 1813)	3						3
*Microlestes maurus* (Sturm, 1827)	49						49
*Microlestes minutulus* (Goeze, 1777)	15				1		16
*Miscodera arctica* (Paykull, 1798)	1						1
*Notiophilus germinyi* (Fauvel, 1863)	1						1
*Notiophilus palustris* (Duftschmid, 1812)	12						12
*Omophron limbatum* (Fabricius, 1777)						1	1
*Oodes helopioides* (Fabricius, 1792)	12				4		16
*Ophonus azureus* (Fabricius, 1775)	17						17
! *Ophonus laticollis* (Mannerheim, 1825)	1						1
*Ophonus puncticollis* (Paykull, 1798)	15				1		16
*Ophonus rufibarbis* (Fabricius, 1792)	30						30
*Ophonus stictus* (Stephens, 1828)	16						16
*Oxypselaphus obscurus* (Herbst, 1784)	6				1		7
*Panagaeus bipustulatus* (Fabricius, 1775)	27						27
*Paradromius linearis* (G.-A. Olivier, 1795)	1				1	1	3
*Patrobus atrorufus* (Strøm, 1768)	7						7
*Poecilus cupreus* (Linnaeus, 1758)	42						42
*Poecilus lepidus* (Leske, 1785)	11						11
*Poecilus versicolor* (Sturm, 1824)	387				13		400
*Pterostichus anthracinus* (Illiger, 1798)	76				1		77
*Pterostichus diligens* (Sturm, 1824)	30						30
*Pterostichus gracilis* (Dejean, 1828)	7						7
*Pterostichus melanarius* (Illiger, 1798)	721				2		723
*Pterostichus minor* (Gyllenhal, 1827)	15				2		17
*Pterostichus niger* (Schaller, 1783)	296						296
*Pterostichus nigrita* (Paykull, 1790)	21						21
*Pterostichus oblongopunctatus* (Fabricius, 1787)	886				1	1	888
*Pterostichus quadrifoveolatus* (Letzner, 1852)	1868						1868
*Pterostichus strenuus* (Panzer, 1796)	20						20
*Pterostichus vernalis* (Panzer, 1796)	11						11
*Sericoda quadripunctata* (De Geer, 1774)	70						70
*Stenolophus mixtus* (Herbst, 1784)	3				1		4
*Stomis pumicatus* (Panzer, 1796)	8						8
*Synuchus vivalis* (Illiger, 1798)	2						2
! *Syntomus obscuroguttatus* (Duftschmid, 1812)					1		1
*Syntomus truncatellus* (Linnaeus, 1761)	3						3
*Tachyta nana* (Gyllenhal, 1810)	1						1
*Trechus secalis* (Paykull, 1790)	9						9
! *Zabrus tenebrioides* (Goeze, 1777)	1						1
**Dytiscidae**							
! *Agabus congener* (Thunberg, 1794)	1						1
! *Dytiscus thianschanicus* (Gschwendtner, 1923)					1		1
*Ilybius ater* (De Geer, 1774)					1		1
*Ilybius erichsoni* (Gemminger and Harold, 1868)					1		1
*Ilybius subtilis* (Erichson, 1837)	1		3		1		5
*Hydaticus continentalis* (J. Balfour-Browne, 1944)	1						1
*Hydaticus seminiger* (De Geer, 1774)	2						2
! *Hydroporus angustatus* (Sturm, 1835			1				1
! *Hydroporus erythrocephalus* (Linnaeus, 1758)	1						1
! *Hydroporus planus* (Fabricius, 1782)					1		1
! *Hydroporus striola* (Gyllenhal, 1826)						7	7
! *Hydroporus tristis* (Paykull, 1798)					2	1	3
! *Rhantus suturellus* (Harris, 1828)					1		1
**Hydrophilidae**							
! *Anacaena lutescens* (Stephens, 1829)	1		2		3		6
! *Cercyon convexiusculus* (Stephens, 1829)	1						1
! *Cercyon marinus* (C.G. Thomson, 1853)					1		1
! *Cercyon sternalis* (Sharp, 1918)	1						1
! *Cercyon tristis* (Illiger, 1801)	1						1
*Cercyon ustulatus* (Preyssler, 1790)					1		1
*Coelostoma orbiculare* (Fabricius, 1775)			1				1
*Enochrus affinis* (Thunberg, 1794)						2	2
*Enochrus coarctatus* (Gredler, 1863)					3		3
*Helochares obscurus* (O.F. Müller, 1776)					2		2
*Hydrobius fuscipes* (Linnaeus, 1758)			1		14	2	17
*Hydrochara caraboides* (Linnaeus, 1758)	1		1		1		3
**Histeridae**							
*Atholus duodecimstriatus* (Schrank, 1781)					1		1
*Hister unicolor* (Linnaeus, 1758)	2		4				6
! *Hypocaccus rufipes* (Kugelann, 1792)					1		1
! *Margarinotus purpurascens* (Herbst, 1791)					1		1
! *Margarinotus striola* (C.R. Sahlberg, 1819)		1					1
! *Plegaderus caesus* (Herbst, 1791)			1				1
! *Saprinus aeneus* (Fabricius, 1775)	6						6
! *Saprinus planiusculus* (Motschulsky, 1849)	6						6
! *Saprinus semistriatus* (L.G. Scriba, 1790)	9						9
! *Saprinus virescens* (Paykull, 1798)					1		1
**Hydraenidae**							
*Ochthebius minimus* (Fabricius, 1792)			1				1
**Leiodidae**							
! *Apocatops nigrita* (Erichson, 1837)	2						2
! *Agathidium rotundatum* (Gyllenhal, 1827)			1				1
! *Amphicyllis globiformis* (C.R. Sahlberg, 1833)			1				1
*Amphicyllis globus* (Fabricius, 1792)			1				1
*Anisotoma castanea* (Herbst, 1791)		1	1				2
*Anisotoma humeralis* (Fabricius, 1791)			2				2
*Anisotoma orbicularis* (Herbst, 1791)			4				4
! *Catops nigricans* (Spence, 1813)	1				1		2
! *Choleva agilis* (Illiger, 1798)	2						2
! *Choleva spinipennis* (Reitter, 1890)	2						2
! *Colenis immunda* (Sturm, 1807)					2		2
! *Cyrtusa subtestacea* (Gyllenhal, 1813)					1		1
! *Fissocatops westi* (Krogerus, 1931)					1		1
! *Leiodes obesa* (W.L.E. Schmidt, 1841)					1		1
! *Leiodes oblonga* (Erichson, 1845)			1				1
! *Leiodes ruficollis* (J. Sahlberg, 1898)					1		1
*Liodopria serricornis* (Gyllenhal, 1813)			1				1
*Sciodrepoides fumatus* (Spence, 1813)			1				1
*Sciodrepoides watsoni* (Spence, 1813)	3		2		8		13
**Staphylinidae**							
*Aleochara brevipennis* (Gravenhorst, 1806)			1			1	2
*Anthobium atrocephalum* (Gyllenhal, 1827)	36		1				37
! *Arpedium brachypterum* (Gravenhorst, 1802)					1		1
*Arpedium quadrum* (Gravenhorst, 1806)	8						8
! *Atheta pallidicornis* (C.G. Thomson, 1856)	25						25
*Bisnius fimetarius* (Gravenhorst, 1802)							
! *Bisnius nitidulus* (Gravenhorst, 1802)	5						5
*Bisnius subuliformis* (Gravenhorst, 1802)			1				1
*Bolitochara pulchra* (Gravenhorst, 1806)	2						2
! *Brachygluta fossulata* (Reichenbach, 1816)	2						2
! *Carphacis striatus* (G.-A. Olivier, 1795)	1						1
! *Deliphrum tectum* (Paykull, 1789)	1						1
*Dendroxena quadrimaculata* (Scopoli, 1771)		8	2				10
*Drusilla canaliculata* (Fabricius, 1787)	9				2		11
! *Encephalus complicans* (Stephens, 1832)	1						1
! *Fagniezia impressa* (Panzer, 1803)	10						10
*Geostiba circellaris* (Gravenhorst, 1806)	1						1
*Ilyobates nigricollis* (Paykull, 1800)	1						1
*Ischnosoma splendidum* (Gravenhorst, 1806)	2						2
*Lathrobium impressum* (Heer, 1841)	1						1
*Lordithon lunulatus* (Linnaeus, 1761)			1		8		9
*Lordithon pulchellus* (Mannerheim, 1830)					4		4
*Necrodes littoralis* (Linnaeus, 1758)		7					7
! *Neuraphes angulatus* (P.W.J. Müller and Kunze, 1822)			1				1
*Nicrophorus humator* (Gleditsch, 1767)	1	7					8
*Nicrophorus interruptus* (Stephens, 1830)	8	7					15
*Nicrophorus investigator* (Zetterstedt, 1824)	5	3					8
*Nicrophorus vespillo* (Linnaeus, 1758)	56	1	1		4	2	64
*Nicrophorus vespilloides* (Herbst, 1783)	19	3	99		1	3	125
*Mycetoporus lepidus* (Gravenhorst, 1806)	1						1
*Oiceoptoma thoracicum* (Linnaeus, 1758)	37	16	4		1	7	65
*Olophrum assimile* (Paykull, 1800)	30				7		37
*Ontholestes murinus* (Linnaeus, 1758)			1				1
*Ochthephilum fracticorne* (Paykull, 1800)	6						6
*Ocypus fulvipennis* (Erichson, 1840)	1						1
*Ocypus nitens* (Schrank, 1781)	20						20
*Ocypus picipennis* (Fabricius, 1792)	1						1
*Omalium caesum* (Gravenhorst, 1806)	1						1
*Oxypoda abdominalis* (Mannerheim, 1830)	16						16
*Oxyporus maxillosus* (Fabricius, 1793)			1				1
*Oxyporus rufus* (Linnaeus, 1758)	1				3		4
! *Oxytelus sculptus* (Gravenhorst, 1806)						1	1
*Paederus littoralis* (Gravenhorst, 1802)	1						1
*Parabolitobius formosus* (Gravenhorst, 1806)	4						4
*Pella cognata* (Märkel, 1842)	13						13
! *Pella limbata* (Paykull, 1789)	18						18
*Philonthus fumarius* (Gravenhorst, 1806)						1	1
*Philonthus micans* (Gravenhorst, 1802)	1					2	3
! *Philonthus micantoides* (Benick and Lohse, 1956)						1	1
! *Philonthus umbratilis* (Gravenhorst, 1802)						1	1
*Phosphuga atrata* (Linnaeus, 1758)	25		1		4		30
! *Platydracus fulvipes* (Scopoli, 1763)	16						16
! *Pselaphus heisei* (Herbst, 1791)	2						2
! *Rabigus tenuis* (Fabricius, 1792)	1						1
*Rugilus rufipes* (Germar, 1836)			1				1
! *Scaphidium quadrimaculatum* (G.-A. Olivier, 1790)			1				1
*Scaphisoma assimile* (Erichson, 1845)			2				2
! *Scaphisoma subalpinum* (Reitter, 1880)			1				1
! *Scydmaenus hellwigii* (Herbst, 1791)			2				2
*Sepedophilus marshami* (Stephens, 1832)	10		1				11
*Silpha carinata* (Herbst, 1783)	133		7		4		144
*Silpha obscura* (Linnaeus, 1758)	305				5		310
*Staphylinus erythropterus* (Linnaeus, 1758)	42						42
! *Stenichnus collaris* (P.W.J. Müller and Kunze, 1822)	6						6
! *Stenichnus godarti* (Latreille, 1806)			1				1
*Stenus clavicornis* (Scopoli, 1763)	2						2
! *Stenus fulvicornis* (Stephens, 1833)						1	1
*Stenus lustrator* (Erichson, 1839)	1						1
*Tachinus bipustulatus* (Fabricius, 1793)		1					1
! *Tetartopeus rufonitidus* (Reitter, 1909)	1						1
*Thanatophilus dispar* (Herbst, 1793)	1						1
*Thanatophilus sinuatus* (Fabricius, 1775)	12				1		13
! *Quedius dilatatus* (Fabricius, 1787)		23	1				24
*Quedius fuliginosus* (Gravenhorst, 1802)	1						1
*Xantholinus longiventris* (Heer, 1839)	3						3
*Xantholinus tricolor* (Fabricius, 1787)	23						23
*Zyras collaris* (Paykull, 1800)	1						1
**Trogidae**							
*Trox sabulosus* (Linnaeus, 1758)	35						35
! *Trox scaber* (Linnaeus 1767)	4						4
**Lucanidae**							
*Platycerus caraboides* (Linnaeus, 1758)	35		5		1		41
*Sinodendron cylindricum* (Linnaeus, 1758)	1	1	2				4
**Geotrupidae**							
*Anoplotrupes stercorosus* (Scriba, 1791)	149		26			5	180
! *Odonteus armiger* (Scopoli, 1772)					2		2
**Scarabaeidae**							
*Acrossus depressus* (Kugelann, 1792)			1				1
*Acrossus rufipes* (Linnaeus, 1758)			7				7
*Agoliinus nemoralis* (Erichson 1848)			1				1
*Amphimallon solstitiale* (Linnaeus, 1758)					4	1	5
*Anomala dubia* (Scopoli, 1763)					1	1	2
*Cetonia aurata* (Linnaeus, 1758)		293				28	321
*Gnorimus variabilis* (Linnaeus, 1758) **		2					2
*Hoplia parvula* (Krynicki, 1832)					2	9	11
! *Maladera holosericea* (Scopoli, 1772)	5				1	1	7
*Melolontha hippocastani* Fabricius, 1801	26	1	5		6	18	56
*Onthophagus ovatus* (Linnaeus, 1767)	42				11		53
*Onthophagus nuchicornis* (Linnaeus, 1758)	1						1
*Oryctes nasicornis* (Linnaeus, 1758)					1	2	3
*Oxythyrea funesta* (Poda von Neuhaus, 1761)					1	42	43
*Phyllopertha horticola* (Linnaeus, 1758)	1		2		3	5	11
*Protaetia cuprea* (Fabricius, 1775)	1	108	2		1	6	118
*Protaetia fieberi* (Kraatz, 1880) *^,^**		74				3	77
*Protaetia marmorata* (Fabricus, 1792) **		448	3			12	463
*Protaetia speciosissima* (Scopoli, 1786) *^,^**		10					10
*Serica brunnea* (Linnaeus, 1758)			31	1	2	8	42
*Trichius fasciatus* (Linnaeus, 1758)	1				1	37	39
*Valgus hemipterus* (Linnaeus, 1758)					1	1	2
**Scirtidae**							
! *Contacyphon kongsbergensis* (Munster, 1923)						2	2
! *Contacyphon laevipennis* (Tournier, 1868)						1	1
! *Contacyphon ochraceus* (Stephens, 1830)			2			8	10
*Contacyphon padi* (Linnaeus, 1758)			1	2		3	6
*Contacyphon pubescens* (Fabricius, 1792)			2			1	3
! *Contacyphon punctipennis* (Sharp, 1872)			1				1
*Contacyphon variabilis* (Thunberg, 1785)			1				1
*Microcara testacea* (Linnaeus,1767)		2	1				3
! *Scirtes hemisphaericus* (Linnaeus, 1758)						7	7
*Scirtes orbicularis* (Panzer, 1793)						1	1
**Eucinetidae**							
! *Eucinetus haemorrhoidalis* (Germar, 1818)	1		1		2		4
**Buprestidae**							
*Agrilus angustulus* (Illiger, 1803)						2	2
*Agrilus cuprescens* (Ménétriés, 1832)						1	1
! *Agrilus hyperici* (Creutzer, 1799)					2		2
*Agrilus sulcicollis* (Lacordaire, 1835)			3				3
*Anthaxia quadripunctata* (Linnaeus, 1758)					11	1	12
! *Coraebus elatus* (Fabricius, 1787)					1		1
! *Cylindromorphus filum* (Gyllenhal, 1817)					3		3
*Dicerca alni* (Fischer von Waldheim, 1824)			1				1
! *Trachys fragariae* (Brisout de Barneville, 1874)					2		2
*Trachys minutus* (Linnaeus, 1758)						30	30
**Byrrhidae**							
! *Byrrhus arietinus* (Steffahny, 1842)	4						4
! *Byrrhus geminatus* (LeConte, 1854)	107						107
! *Byrrhus fasciatus* (Forster, 1771)	15						15
*Byrrhus pilula* (Linnaeus, 1758)	11						11
*Byrrhus pustulatus* (Forster, 1771)	2						2
! *Curimopsis paleata* (Erichson, 1846)	1						1
*Cytilus sericeus* (Forster, 1771)	2						2
! *Morychus aeneus* (Fabricius, 1775)	4						4
! *Porcinolus murinus* (Fabricius, 1794)					1		1
**Heteroceridae**							
*Heterocerus fenestratus* (Thunberg, 1784)					2		2
! *Heterocerus fusculus* (Kiesenwetter, 1843)					2		2
**Eucnemidae**							
! *Hylis procerulus* (Mannerheim, 1823)			2				2
! *Isorhipis marmottani* (Bonvouloir, 1871)			1				1
! *Melasis buprestoides* (Linnaeus, 1761)	1						1
! *Microrhagus pygmaeus* (Fabricius, 1792)			5				5
*Otho sphondyloides* (Germar, 1818)			1				1
**Throscidae**							
! *Aulonothroscus laticollis* (Rybinski, 1897)			1				1
*Trixagus dermestoides* (Linnaeus, 1767)	17		29		1	1	48
**Elateridae**							
*Agriotes lineatus* (Linnaeus, 1767)					3	4	7
*Agriotes obscurus* (Linnaeus, 1758)	8				20	8	36
*Agriotes sputator* (Linnaeus, 1758)	206				82	5	293
*Agrypnus murinus* (Linnaeus, 1758)	47	17	1	1	10	13	89
*Ampedus balteatus* (Linnaeus, 1758)	2	1	1				4
! *Ampedus cinnabarinus* (Eschscholtz, 1829)		22					22
*Ampedus nigrinus* (Herbst, 1784)	2						2
! *Ampedus nigroflavus* (Goeze, 1777)		2	1				3
*Ampedus pomonae* (Stephens, 1830)		3	2				5
! *Ampedus pomorum* (Herbst, 1784)		34	4				38
! *Ampedus praeustus* (Fabricius, 1792)					1		1
*Ampedus sanguinolentus* (Schrank, 1776)		30	1			1	32
! *Ampedus sanguineus* (Linnaeus, 1758)		2				1	3
*Athous haemorrhoidalis* (Fabricius, 1801)			2	2		8	12
*Athous subfuscus* (O.F. Müller, 1764)	9		6			3	18
*Athous vittatus* (Fabricius, 1792)		3	47	1			51
! *Cardiophorus ruficollis* (Linnaeus, 1758)	4						4
*Cidnopus aeruginosus* (G.-A. Olivier, 1790)			18				18
*Dalopius marginatus* (Linnaeus, 1758)		2	57	1			60
*Danosoma conspersum* (Gyllenhal, 1808)			3				3
*Denticollis linearis* (Linnaeus, 1758)			2				2
*Dicronychus cinereus* (Herbst, 1784)					1		1
*Dicronychus equiseti* (Herbst, 1784)					1		1
! *Elater ferrugineus* Linnaeus, 1758) *		1					1
*Hemicrepidius niger* (Linnaeus, 1758)		2					2
*Lacon lepidopterus* (Panzer, 1800)			1				1
! *Limonius minutus* (Linnaeus, 1758)		1	3	1	2		7
! *Melanotus castanipes* (Paykull, 1800)			1				1
*Melanotus villosus* (Geoffroy, 1785)		3	7				10
*Mosotalesus impressus* (Fabricius, 1792)	4						4
*Mosotalesus nigricornis* (Panzer, 1799)		1				10	11
*Oedostethus quadripustulatus* (Fabricius, 1792)					17		17
*Pristilophus cruciatus* (Linnaeus, 1758)			45			2	47
*Prosternon tessellatum* (Linnaeus, 1758)	60	132	4		8	15	219
*Selatosomus aeneus* (Linnaeus, 1758)	5		3		6	1	15
*Selatosomus latus* (Fabricius, 1801)	7				3	2	12
**Lycidae**							
! *Dictyoptera aurora* (Herbst, 1784)	1						1
*Lygistopterus sanguineus* (Linnaeus, 1758)	1		2			58	61
**Lampyridae**							
*Lampyris noctiluca* (Linnaeus, 1758)	5		7				12
**Cantharidae**							
! *Cantharis figurata* (Mannerheim, 1843)		1		4		1	6
*Cantharis flavilabris* (Fallén, 1807)		1		17	25	9	52
*Cantharis lateralis* (Linnaeus, 1758)						37	37
*Cantharis livida* (Linnaeus, 1758)		167				3	170
*Cantharis obscura* (Linnaeus, 1758)	2	1					3
*Cantharis nigricans* (O.F. Müller, 1776)		25	4	2	6		37
*Cantharis pallida* (Goeze, 1777)						2	2
! *Cantharis paludosa* (Fallén, 1807)				1			1
*Cantharis pellucida* (Fabricius, 1792)		3	1	3	1	2	10
*Cantharis rufa* (Linnaeus, 1758)		1			5	8	14
*Cantharis rustica* (Fallén, 1807)	4				1	13	18
*Malthinus flaveolus* (Herbst, 1786)					4		4
! *Malthodes dimidiaticollis* (Rosenhauer, 1847)					1		1
! *Malthodes fuscus* (Waltl, 1838)	2						2
*Malthodes minimus* (Linnaeus, 1758)					1		1
*Rhagonycha fulva* (Scopoli, 1763)		8			1	18	27
! *Rhagonycha fugax* Mannerheim, 1843)						1	1
*Rhagonycha lignosa* (O.F. Müller, 1764)	1		2		6	4	13
! *Rhagonycha nigripes* (W. Redtenbacher, 1842)						1	1
*Rhagonycha testacea* (Linnaeus, 1758)				3			3
*Silis ruficollis* (Fabricius, 1775)						1	1
**Dermestidae**							
*Anthrenus museorum* (Linnaeus, 1761)			1				1
*Attagenus schaefferi* (Herbst, 1792)		8				17	25
*Attagenus smirnovi* (Zhantiev, 1973)						1	1
! *Ctesias serra* (Fabricius, 1792)		13					13
*Dermestes laniarius* (Illiger, 1801)	576			1	41		618
! *Dermestes lardarius* (Linnaeus, 1758)		1					1
*Dermestinus murinus* (Linnaeus, 1758)	75	7					82
*Megatoma undata* (Linnaeus, 1758)			1				1
! *Trogoderma glabrum* (Herbst, 1783)		1					1
**Ptinidae**							
*Cacotemnus rufipes* (Fabricius, 1792)		1	1				2
! *Caenocara affine* (Sturm, 1837)			1		1		2
! *Dorcatoma chrysomelina* (Sturm, 1837)			2				2
! *Dorcatoma dresdensis* (Herbst, 1792)		1	1				2
! *Dorcatoma punctulata* (Mulsant and Rey, 1864)			1				1
! *Dorcatoma robusta* (A. Strand, 1938)			2				2
! *Dorcatoma setosella* (Mulsant and Rey, 1864)			2				2
*Hadrobregmus pertinax* (Linnaeus, 1758)		1	1				2
! *Priobium carpini* (Herbst, 1793)			2				2
! *Ptilinus fuscus* (Geoffroy, 1785)			1				1
! *Ptinus fur* (Linnaeus, 1758)	9						9
! *Ptinus raptor* (Sturm, 1837)					1		1
*Ptinus rufipes* (G.-A. Olivier, 1790)		1	6				7
**Lymexylidae**							
*Elateroides dermestoides* (Linnaeus, 1761)			1				1
**Byturidae**							
! *Byturus ochraceus* (L.G. Scriba, 1790)					19	7	26
**Trogossitidae**							
! *Grynocharis oblonga* (Linnaeus, 1758)			1				1
**Cleridae**							
! *Necrobia violacea* (Linnaeus, 1758)			1				1
! *Thanasimus femoralis* (Zetterstedt, 1828)			1				1
*Thanasimus formicarius* (Linnaeus, 1758)	1	6				10	17
*Tillus elongatus* (Linnaeus, 1758)			1				1
*Trichodes apiarius* (Linnaeus, 1758)						35	35
**Melyridae**							
! *Anthocomus equestris* (Fabricius, 1781)					1		1
! *Apalochrus femoralis* Erichson, 1840)					7	2	9
! *Charopus flavipes* (Paykull, 1798)					1		1
*Cordylepherus viridis* (Fabricius, 1787)				6	34	14	54
*Dasytes niger* (Linnaeus, 1761)		3	12	1	18	34	68
! *Dasytes plumbeus* (Müller, 1776)		3	2			2	7
*Dolichosoma lineare* (P. Rossi, 1794)					2	38	40
*Malachius bipustulatus* (Linnaeus, 1758)		1	2	2	1	46	52
**Sphindidae**							
*Sphindus dubius* (Gyllenhal, 1808)	1						1
**Erotylidae**							
! *Combocerus glaber* (Schaller, 1783)	12						12
! *Dacne bipustulata* (Thunberg, 1781)		1	4				5
! *Triplax aenea* (Schaller, 1783)	1						1
! *Triplax lepida* (Faldermann, 1837)			1				1
! *Triplax rufipes* (Fabricius, 1787)	4		3		6		13
*Triplax russica* (Linnaeus, 1758)			4			1	5
*Tritoma bipustulata* (Fabricius, 1775)			1				1
**Monotomidae**							
! *Rhizophagus bipustulatus* (Fabricius, 1792)			1				1
! *Rhizophagus fenestralis* (Linnaeus, 1758)		6			1		7
**Kateretidae**							
! *Brachypterolus pulicarius* (Linnaeus, 1758)					1		1
! *Brachypterus urticae* (Fabricius, 1792)						3	3
**Nitidulidae**							
*Amphotis marginata* (Fabricius, 1781)					1		1
*Carpophilus hemipterus* (Linnaeus, 1758)		1					1
*Carpophilus marginellus* (Motschulsky, 1858)		4					4
*Cryptarcha strigata* (Fabricius, 1787)		1183	10				1193
*Cryptarcha undata* (G.-A. Olivier, 1790)		65					65
*Cyllodes ater* (Herbst, 1792)			4				4
*Epuraea aestiva* (Linnaeus, 1758)		1					1
*Epuraea biguttata* (Thunberg, 1784)		39	1				40
*Epuraea guttata* (G.-A. Olivier, 1811)		35	2				37
*Epuraea longula* (Erichson, 1845)			1				1
*Epuraea marseuli* (Reitter, 1873)			1				1
*Glischrochilus grandis* (Tournier, 1872)		302	43				345
*Glischrochilus hortensis* (Geoffroy, 1785)		105	2				107
*Glischrochilus quadriguttatus* (Fabricius, 1777)		3					3
*Glischrochilus quadripunctatus* (Linnaeus, 1758)	1	46					47
*Glischrochilus quadrisignatus* (Say, 1835)		32					32
*Meligethes pedicularius* (Gyllenhal, 1808)						1	1
*Meligethes viridescens* (Fabricius, 1787)						1	1
*Pityophagus ferrugineus* (Linnaeus, 1761)	1						1
*Pocadius ferrugineus* (Fabricius, 1775)			1				1
*Soronia grisea* (Linnaeus, 1758)		825	1				826
*Soronia punctatissima* (Illiger, 1794)		2					2
*Thalycra fervida* (G.-A. Olivier, 1790)			2				2
**Cryptophagidae**							
! *Telmatophilus caricis* (Olivier, 1790)						1	1
**Silvanidae**							
! *Ahasverus advena* (Waltl, 1834)		1					1
**Cucujidae**							
! *Pediacus depressus* (Herbst, 1797)			18				18
**Phalacridae**							
! *Olibrus affinis* (Sturm, 1807)						1	1
*Olibrus bicolor* (Fabricius, 1792)						1	1
*Olibrus millefolii* (Paykull, 1800)						41	41
*Phalacrus caricis* (Sturm, 1807)						3	3
**Laemophloeidae**							
! *Lathropus sepicola* (P.W.J. Müller, 1821)			1				1
**Cerylonidae**							
! *Cerylon ferrugineum* (Stephens, 1830)			1				1
! *Cerylon histeroides* (Fabricius, 1792)			1				1
**Latridiidae**							
! *Corticaria polypori* (J.R. Sahlberg, 1900)	1						1
! *Cortinicara gibbosa* (Herbst, 1793)			4		10		14
! *Enicmus rugosus* (Herbst, 1793)			6		1		7
! *Latridius porcatus* (Herbst, 1793)			3				3
! *Stephostethus angusticollis* (Gyllenhal, 1827)			2				2
*Stephostethus lardarius* (De Geer, 1775)			1				1
**Anamorphidae**							
! *Symbiotes gibberosus* (Lucas, 1846)			1				1
**Endomychidae**							
*Dapsa horvathi* (Csiki, 1901)	7				1		8
! *Endomychus coccineus* (Linnaeus, 1758)			3				3
*Mycetina cruciata* (Schaller, 1783)	1						1
**Coccinellidae**							
*Adalia bipunctata* (Linnaeus, 1758)						8	8
*Adalia decempunctata* (Linnaeus, 1758)				1			1
*Anatis ocellata* (Linnaeus, 1758)		1			1	3	5
*Anisosticta novemdecimpunctata* (Linnaeus, 1758)						15	15
*Calvia decemguttata* (Linnaeus, 1767)			1	2		12	15
*Calvia quatuordecimguttata* (Linnaeus, 1758)	1			6		1	8
*Ceratomegilla notata* (Laicharting, 1781)	1				3		4
*Chilocorus renipustulatus* (L.G. Scriba, 1791)			1		1		2
*Coccidula rufa* (Herbst, 1783)						2	2
*Coccinella hieroglyphica* (Linnaeus, 1758)						1	1
*Coccinella magnifica* (L. Redtenbacher, 1843)						5	5
*Coccinella septempunctata* (Linnaeus, 1758)	3		1		7	70	81
*Coccinella quinquepunctata* (Linnaeus, 1758)					1		1
*Coccinula quatuordecimpustulata* (Linnaeus, 1758)	2				17	11	30
*Exochomus quadripustulatus* (Linnaeus, 1758)		1					1
*Halyzia sedecimguttata* (Linnaeus, 1758)		1	1				2
*Harmonia axyridis* (Pallas, 1773)	1	2		1		7	11
*Harmonia quadripunctata* (Pontoppidan, 1763)		1					1
! *Hyperaspis concolor* (Suffrian, 1843)					1		1
! *Hyperaspis pseudopustulata* (Mulsant, 1853)						1	1
*Hippodamia tredecimpunctata* (Linnaeus, 1758)					2	3	5
*Hippodamia variegata* (Goeze, 1777)					1	13	14
! *Nephus redtenbacheri* (Mulsant, 1846)					1		1
! *Oenopia conglobata* (Linnaeus, 1758)					2		2
*Platynaspis luteorubra* (Goeze, 1777)	2				2	1	5
*Propylea quatuordecimpunctata* (Linnaeus, 1758)	1			9	16	10	36
*Psyllobora vigintiduopunctata* (Linnaeus, 1758)			1	1	26	5	33
! *Scymnus apetzi* (Mulsant, 1846)					4		4
! *Scymnus femoralis* (Gyllenhal, 1827)						1	1
! *Scymnus ferrugatus* (Moll, 1785)				1			1
*Scymnus frontalis* (Fabricius, 1787)					27		27
*Scymnus haemorrhoidalis* (Herbst, 1797)				1	5		6
*Scymnus rubromaculatus* (Goeze, 1778)					1		1
! *Stethorus pusillus* (Herbst, 1797)					3		3
*Subcoccinella vigintiguatuorpunctata* (Linnaeus, 1758)						5	5
*Tytthaspis gebleri* (Mulsant, 1850)					20		20
*Tytthaspis sedecimpunctata* (Linnaeus, 1761)				1	1		2
**Mycetophagidae**							
*Litargus connexus* (Geoffroy, 1785)		6	3				9
! *Mycetophagus ater* (Reitter, 1879)			5				5
*Mycetophagus piceus* (Fabricius, 1777)			2				2
*Mycetophagus quadripustulatus* (Linnaeus, 1761)			12				12
! *Triphyllus bicolor* (Fabricius, 1777)			1				1
**Ciidae**							
! *Cis comptus* (Gyllenhal, 1827)			1				1
! *Orthocis alni* (Gyllenhal, 1813)			1				1
**Melandryidae**							
! *Melandrya barbata* (Fabricius, 1787) *			2				2
! *Melandrya dubia* (Schaller, 1783)			3				3
! *Orchesia micans* (Panzer, 1793)		1					1
! *Osphya bipunctata* (Fabricius, 1775)			4				4
! *Phloiotrya subtilis* (Reitter, 1897)		1					1
! *Phryganophilus ruficollis* (Fabricius, 1798)			1				1
! *Xylita laevigata* (Hellenius, 1786)					1		1
**Zopheridae**							
*Bitoma crenata* (Fabricius, 1775)					1		1
*Orthocerus clavicornis* (Linnaeus, 1758)					1		1
*Synchita humeralis* (Fabricius, 1792)			1				1
**Mordellidae**							
! *Mordella brachyura* (Mulsant, 1856)						2	2
! *Mordella holomelaena* (Apfelbeck, 1914)						10	10
! *Mordellistena humeralis* (Linnaeus, 1758)	1						1
! *Mordellistena pygmaeola* (Ermisch, 1956)						3	3
*Mordellistena pumila* (Gyllenhal, 1810)						20	20
! *Mordellistena variegata* (Fabricius, 1798)			1				1
! *Variimorda basalis* (A. Costa, 1854)						5	5
! *Variimorda villosa* (Schrank von Paula, 1781)		1	1		1	2	5
**Tenebrionidae**							
! *Blaps lethifera* (Marsham, 1802)	99						99
*Bolitophagus reticulatus* (Linnaeus, 1767)	1					1	2
*Crypticus quisquilius* (Linnaeus, 1761)	413				3		416
! *Cteniopus sulphuripes* (Germar, 1823)					4		4
! *Isomira murina* (Linnaeus, 1758)	1					8	9
*Lagria hirta* (Linnaeus, 1758)	9		3	6	21	69	108
! *Mycetochara flavipes* (Fabricius, 1792)			14				14
! *Nalassus brevicollis* (Krynicki, 1832)	1						1
*Neomidia haemorrhoidalis* (Fabricius, 1787)			1				1
*Opatrum sabulosum* (Linnaeus, 1761)	278				64		342
! *Palorus depressus* (Fabricius, 1790)			1				1
! *Pedinus femoralis* (Linnaeus, 1767)	130						130
*Pseudocistela ceramboides* (Linnaeus, 1758)	1				1		2
*Scaphidema metallica* (Fabricius, 1792)	2						2
*Upis ceramboides* (Linnaeus, 1758)	8		1			3	12
**Oedemeridae**							
! *Anogcodes melanurus* (Fabricius, 1787)						1	1
*Chrysanthia geniculata* (W.L.E. Schmidt, 1846)			2			11	13
*Chrysanthia viridissima* (Linnaeus, 1758)		3	2		1	47	53
*Oedemera femorata* (Scopoli, 1763)		1	1		5	21	28
*Oedemera lurida* (Marsham, 1802)					1	14	15
! *Oedemera podagrariae* (Linnaeus, 1767)						10	10
! *Oedemera rostralis* (Reitter, 1885)					6	13	19
! *Oedemera subrobusta* (Nakane, 1954)					2	4	6
*Oedemera virescens* (Linnaeus, 1767)					30	11	41
**Meloidae**							
*Lytta vesicatoria* (Linnaeus, 1758)	1						1
*Meloe proscarabaeus* (Linnaeus, 1758)	14						14
*Meloe variegatus* (Donovan, 1793) **	1						1
*Meloe violaceus* (Marsham, 1802)	8						8
*Mylabris pusilla* (G.-A. Olivier, 1811)	1				19		20
**Pyrochroidae**							
*Pyrochroa coccinea* (Linnaeus, 1761)		8					8
*Schizotus pectinicornis* (Linnaeus, 1758)		1	2	1			4
**Salpingidae**							
! *Salpingus planirostris* (Fabricius, 1787)			2				2
! *Salpingus ruficollis* (Linnaeus, 1761)			1				1
**Anthicidae**							
*Anthicus antherinus* (Linnaeus, 1761)					2		2
*Notoxus monoceros* (Linnaeus, 1761)	1				6	2	9
! *Omonadus floralis* (Linnaeus, 1758)						1	1
**Aderidae**							
*Anidorus nigrinus* (Germar, 1842)					12		12
! *Phytobaenus amabilis* (R.F. Sahlberg, 1834)					1		1
**Scraptiidae**							
! *Anaspis frontalis* (Linnaeus, 1758)		1					1
*Anaspis pulicaria* (A. Costa, 1854)						3	3
! *Anaspis subtilis* (Hampe, 1871)						1	1
! *Anaspis thoracica* (Linnaeus, 1758)			2		1		3
**Cerambycidae**							
! *Agapanthia cardui* (Linnaeus, 1767)					1		1
*Agapanthia villosoviridescens* (De Geer, 1775)						2	2
! *Alosterna tabacicolor* (De Geer, 1775)			1	1	1	7	10
*Anastrangalia reyi* (L. Heyden, 1889)						4	4
*Anastrangalia sanguinolenta* (Linnaeus, 1761)			2			2	4
*Anoplodera sexguttata* (Fabricius, 1775)						1	1
*Arhopalus rusticus* (Linnaeus, 1758)	1						1
*Aromia moschata* (Linnaeus, 1758)		68					68
*Cortodera femorata* (Fabricius, 1787)		1	1				2
*Deilus fugax* (G.-A. Olivier, 1790)					1		1
*Dinoptera collaris* (Linnaeus, 1758)		1				22	23
*Dorcadion holosericeum* (Krynicki, 1832)	47						47
*Euracmaeops marginatus* (Fabricius, 1781)		1					1
*Lamia textor* (Linnaeus, 1758)	3						3
*Leiopus linnei* (Wallin, Nylander, and Kvamme, 2009)		1	1				2
! *Leptura aethiops* (Poda von Neuhaus, 1761)			1				1
*Leptura quadrifasciata* (Linnaeus, 1758)	1	97	6		1	29	134
*Leptura thoracica* (Creutzer, 1799) **		55					55
*Lepturalia nigripes* (De Geer, 1775)	1	6					7
*Lepturobosca virens* (Linnaeus, 1758)			2			10	12
*Mesosa myops* (Dalman, 1817)		2				2	4
*Molorchus minor* (Linnaeus, 1758)		3				1	4
! *Molorchus umbellatarum* (Schreber, 1759)		3	1				4
*Monochamus galloprovincialis pistor* (Olivier, 1795)			1				1
*Necydalis major* (Linnaeus, 1758) **		50					50
! *Nivellia sanguinosa* (Gyllenhal, 1827)			1				1
! *Obrium cantharinum* (Linnaeus, 1767)		14					14
*Pachyta quadrimaculata* (Linnaeus, 1758)						3	3
*Phymatodes testaceus* (Linnaeus, 1758)		90					90
*Phytoecia pustulata* (Schrank, 1776)					4		4
*Plagionotus arcuatus* (Linnaeus, 1758)		1					1
*Plagionotus detritus* (Linnaeus, 1758)		104					104
*Pogonocherus fasciculatus* (De Geer, 1775)	1						1
*Prionus coriarius* (Linnaeus, 1758)	2		3			1	6
*Pseudovadonia livida bicarinata* (N. Arnold, 1869)					1	3	4
*Purpuricenus kaehleri* (Linnaeus, 1758) **		9					9
*Pyrrhidium sanguineum* (Linnaeus, 1758)		1					1
*Rhagium inquisitor* (Linnaeus, 1758)	5	4					9
*Rhagium mordax* (De Geer, 1775)	1	97	3		1	57	159
*Rhagium sycophanta* (Schrank, 1781)		2					2
! *Rutpela maculata* (Poda von Neuhaus, 1761)						15	15
*Spondylis buprestoides* (Linnaeus, 1758)						3	3
*Stenurella bifasciata* (O.F. Müller, 1776)			1			16	17
*Stenurella melanura* (Linnaeus, 1758)	1		2			35	38
! *Stictoleptura maculicornis* (De Geer, 1775)			1			2	3
*Stictoleptura rubra* (Linnaeus, 1758)			3		1	10	14
*Strangalia attenuata* (Linnaeus, 1758)		1	1			41	43
*Tetrops praeustus* (Linnaeus, 1758)	1		1		1		3
! *Trichoferus campestris* (Faldermann, 1835)		1					1
*Xylotrechus antilope* (Schoenherr, 1817)		11					11
*Xylotrechus rusticus* (Linnaeus, 1758)		3				4	7
**Orsodacnidae**							
*Orsodacne cerasi* (Linnaeus, 1758)		1		2			3
**Chrysomelidae**							
*Agelastica alni* (Linnaeus, 1758)						1	1
*Altica brevicollis* (Foudras, 1861)						4	4
! *Altica engstroemi* (J. Sahlberg, 1893)					1		1
! *Altica lythri* (Aubé, 1843)			1				1
*Altica oleracea* (Linnaeus, 1758)			1			4	5
! *Aphthona euphorbiae* (Schrank, 1781)	1				1		2
! *Aphthona lutescens* (Gyllenhal, 1813)					1	16	17
! *Aphthona nigriscutis* (Foudras, 1860)					1		1
! *Aphthona nonstriata* (Goeze, 1777)	1				1		2
! *Batophila fallax* (Weise, 1888)					16		16
*Bromius obscurus* (Linnaeus, 1758)						1	1
*Cassida margaritacea* (Schaller, 1783)					1		1
*Cassida nebulosa* (Linnaeus, 1758)					1	1	2
! *Cassida pannonica* (Suffrian, 1844)					2		2
*Cassida panzeri* (J. Weise, 1907)					1		1
*Cassida prasina* (Illiger, 1798)	1				5	12	18
*Cassida vibex* (Linnaeus, 1767)					1		1
*Cassida viridis* (Linnaeus, 1758)						2	2
*Chaetocnema aridula* (Gyllenhal, 1827)	1				2		3
! *Chaetocnema compressa* (Letzner, 1847)					8		8
! *Chaetocnema concinna* (Marsham, 1802)		3			5	1	9
! *Chaetocnema hortensis* (Geoffroy, 1785)	2	1			23	2	28
*Chaetocnema mannerheimi* (Gyllenhal, 1827)						6	6
! *Chrysolina aurichalcea* (Gebler in Mannerheim, 1825)						1	1
*Chrysolina graminis* (Linnaeus, 1758)						2	2
*Chrysolina fastuosa* (Scopoli, 1763)	1						1
*Chrysolina polita* (Linnaeus, 1758)					2	6	8
*Chrysolina sanguinolenta* (Linnaeus, 1758)	1						1
*Chrysolina staphylaea* (Linnaeus, 1758)	1					1	2
*Chrysolina sturmi* (Westhoff, 1882)	7					9	16
*Chrysolina varians* (Schaller, 1783)	4				4	1	9
*Chrysomela vigintipunctata* (Scopoli, 1763)						3	3
*Coptocephala unifasciata* (Scopoli, 1763)	2						2
*Crepidodera aurata* (Marsham, 1802)			1				1
*Crepidodera aurea* (Geoffroy, 1785)	1					2	3
*Crepidodera fulvicornis* (Fabricius, 1792)	2					1	3
*Cryptocephalus anticus* (Suffrian, 1848)						1	1
*Cryptocephalus bipunctatus* (Linnaeus, 1758)						5	5
*Cryptocephalus bilineatus* (Linnaeus, 1767)					2		2
*Cryptocephalus exiguus* (D.N. Schneider, 1792)					2		2
*Cryptocephalus flavipes* (Fabricius, 1781)	1				1	5	7
*Cryptocephalus fulvus* (Goeze, 1777)						2	2
*Cryptocephalus labiatus* (Linnaeus, 1761)					2		2
*Cryptocephalus laetus* Fabricius, 1792)					4		4
*Cryptocephalus moraei* (Linnaeus, 1758)		1		1	29	5	36
! *Cryptocephalus parvulus* (O.F. Müller, 1776)					8		8
*Cryptocephalus sericeus* (Linnaeus, 1758)					4	3	7
*Cryptocephalus solivagus* (Leonardi and Sassi, 2001)	8	1			7	3	19
! *Derocrepis rufipes* (Linnaeus, 1758)	3				1		4
! *Dibolia cryptocephala* (Koch, 1803)	1						1
! *Dibolia metallica* (Motschulsky, 1845)	15				1		16
*Donacia semicuprea* (Panzer, 1796)				1		1	2
*Donacia thalassina* (Germar, 1811)						1	1
*Galeruca pomonae* (Scopoli, 1763)	1					2	3
*Galeruca tanaceti* (Linnaeus, 1758)	5		3		2		10
*Galerucella calmariensis* (Linnaeus, 1767)						1	1
*Galerucella lineola* (Fabricius, 1781)	2	1			1	30	34
*Galerucella tenella* (Linnaeus, 1761)				1		4	5
*Gastrophysa polygoni* (Linnaeus, 1758)					2	7	9
*Gastrophysa viridula* (De Geer, 1775)						1	1
*Hypocassida subferruginea* (Schrank, 1776)		3	1		13		17
*Labidostomis longimana* (Linnaeus, 1761)	1				4	7	12
*Lema cyanella* (Linnaeus, 1758)		1			1		2
*Lilioceris merdigera* (Linnaeus, 1758)			1				1
*Lochmaea caprea* (Linnaeus, 1758)						7	7
! *Longitarsus anchusae* (Paykull, 1799)	1						1
! *Longitarsus jacobaeae* (C.R. Waterhouse, 1858)					15	3	18
! *Longitarsus luridus* (Scopoli, 1763)					17		17
! *Longitarsus minimus* (Kutschera, 1863)					3		3
! *Longitarsus nigrofasciatus* (Goeze, 1777)					1		1
! *Longitarsus noricus* (Leonardi, 1976)					12		12
! *Longitarsus parvulus* (Paykull, 1799)					3	1	4
*Longitarsus pellucidus* (Foudras, 1860)					1		1
! *Longitarsus succineus* (Foudras, 1860)		53			383		436
*Lythraria salicariae* (Paykull, 1800)	1				1		2
*Neocrepidodera crassicornis* (Faldermann, 1837)						1	1
*Neocrepidodera ferruginea* (Scopoli, 1763)	1				5		6
! *Neocrepidodera motschulskii* (Konstantinov, 1991)	1	3			6		10
*Neocrepidodera transversa* (Marscham, 1802)					3	1	4
*Oulema melanopus* (Linnaeus, 1758)						1	1
! *Pachybrachis fimbriolatus* (Suffrian, 1848)					1		1
*Phratora atrovirens* (Cornelius, 1857)			2			4	6
*Phratora laticollis* (Suffrian, 1851)			2	1		1	4
*Phratora vulgatissima* (Linnaeus, 1758)			1			3	4
*Phyllotreta atra* (Fabricius, 1775)		1			3	2	6
! *Phyllotreta cruciferae* (Goeze, 1777)	3				25		28
! *Phyllotreta flexuosa* (Illiger, 1794)				2			2
*Phyllotreta nemorum* (Linnaeus, 1758)					1		1
! *Phyllotreta nigripes* (Fabricius, 1775)			1				1
! *Phyllotreta tetrastigma* (Comolli, 1837)				2			2
*Phyllotreta undulata* (Kutschera, 1860					2		2
*Phyllotreta vittula* (L. Redtenbacher, 1849)		1			66	3	70
*Plagiodera versicolora* (Laicharting, 1781)		1			6	1	8
*Plagiosterna aenea* (Linnaeus, 1758)						10	10
! *Psylliodes chalcomera* (Illiger, 1807)					1		1
! *Psylliodes napi* (Fabricius, 1792)					1		1
! *Psylliodes picina* (Marsham, 1802)					6		6
*Spermophagus sericeus* (Geoffroy, 1785)					2		2
**Anthribidae**							
*Anthribus nebulosus* (Forster, 1770)			1				1
*Dissoleucas niveirostris* (Fabricius, 1798)			2	1			3
! *Gonotropis dorsalis* (Gyllenhal, 1813)		1					1
*Platystomos albinus* (Linnaeus, 1758)	10		2		1		13
*Tropideres albirostris* (Schaller, 1783)		3					3
**Attelabidae**							
*Apoderus coryli* (Linnaeus, 1758)					1		1
*Byctiscus betulae* (Linnaeus, 1758)			1		1		2
*Neocoenorrhinus germanicus* (Herbst, 1797)					1		1
! *Rhynchites auratus* (Scopoli, 1763)					1		1
**Brentidae**							
! *Aspidapion chalceus* (Marsham, 1802)					1		1
*Aspidapion radiolus* (Marsham, 1802)					1		1
*Betulapion simile* (Kirby, 1811)			1			1	2
*Catapion seniculus* (Kirby, 1808)	1						1
*Ceratapion onopordi* (Kirby, 1808)	1						1
! *Cyanapion columbinum* (Germar, 1817)					1		1
*Diplapion detritum* (Mulsant and Rey, 1859)					1		1
*Eutrichapion facetum* (Gyllenhal, 1839)	1				1		2
! *Ischnopterapion loti* (Kirby, 1808)					1		1
! *Loborhynchapion amethystinum* (Miller, 1857)	1						1
*Mesotrichapion punctirostre* (Gyllenhal, 1839)	1						1
*Nanophyes globiformis* (Kiesenwetter, 1864)					1		1
*Nanophyes marmoratus* (Goeze, 1777)						6	6
*Omphalapion hookerorum* (Kirby, 1808)		1					1
*Oxystoma craccae* (Linnaeus, 1767)					3		3
*Perapion marchicum* (Herbst, 1797)						1	1
*Perapion oblongum* (Gyllenhal, 1839)					1		1
*Perapion violaceum* (Kirby, 1808)					1		1
*Protapion assimile* (Kirby, 1808)	1				1		2
*Protapion apricans* (Herbst, 1797)					2		2
*Protapion fulvipes* (Geoffroy, 1785)	2	2		1	15	1	21
*Protapion interjectum* (Desbrochers des Loges, 1895)	1						1
*Protapion trifolii* (Linnaeus, 1768)	1					1	2
*Protapion varipes* (Germar, 1817)					1		1
*Pseudoperapion brevirostre* (Herbst, 1797)					3	1	4
*Stenopterapion tenue* (Kirby, 1808)						6	6
**Curculionidae**							
! *Anisandrus dispar* (Fabricius, 1792)		30	1				31
*Anoplus plantaris* (Næzén, 1794)		1					1
*Anthonomus pomorum* (Linnaeus, 1758)					1	1	2
*Anthonomus rectirostris* (Linnaeus, 1758)					1		1
*Auleutes epilobii* (Paykull, 1800)					1		1
! *Bagous aliciae* (Cmoluch, 1983)	1						1
*Bothynoderes affinis* (Schrank, 1781)	1						1
*Brachyderes incanus* (Linnaeus, 1758)	1						1
*Brachysomus echinatus* (Bonsdorff, 1785)	15		1		14		30
! *Cathormiocerus aristatus* (Gyllenhal, 1827)	3						3
*Ceutorhynchus typhae* (Herbst, 1795)					1		1
*Cionus montanus* (Wingelmüller, 1914)					1		1
! *Cionus olivieri* (Rosenschoeld, 1838)						1	1
*Cleonis pigra* (Scopoli, 1763)	2				2		4
*Cleopomiarus distinctus* (Boheman, 1845)					9		9
! *Coeliodinus phrymos* (Alonso-Zarazaga and Colonelli, 2017)			1				1
*Curculio glandium* (Marsham, 1802)						1	1
*Curculio nucum* (Linnaeus, 1758)		1					1
! *Curculio venosus* (Gravenhorst, 1807)					1		1
*Cycloderes pilosulus* (Herbst, 1795)	2						2
*Cyphocleonus dealbatus* (Gmelin, 1790)	1						1
*Eusomus ovulum* (Germar, 1823)	5				3	18	26
! *Exomias pellucidus* (Boheman, 1834)	95				2		97
! *Foucartia squamulata* (Herbst, 1795)	4						4
*Glocianus distinctus* (C.N.F. Brisout de Barneville, 1870)					1	6	7
*Glocianus punctiger* (C.R. Sahlberg, 1835)					1		1
*Grypus equiseti* (Fabricius, 1775)	1						1
*Gymnetron melanarium* (Germar, 1821)		1			1		2
*Hylastes brunneus* (Erichson, 1836)	26						26
*Hylastes opacus* (Erichson, 1836)	21						21
*Hylobius abietis* (Linnaeus, 1758)	1785		3			69	1857
*Hylobius transversovittatus* (Goeze, 1777)					1		1
*Hypera arator* (Linnaeus, 1758)					1		1
*Hypera conmaculata* (Herbst, 1795)					1		1
! *Hypera interruptovittata* (Desbrochers des Loges, 1875)	1						1
! *Hypera fornicata* (Penecke, 1928)	1				1		2
*Hypera meles* (Fabricius, 1792)	14				1		15
*Hypera miles* (Paykull, 1792)	2				5	1	8
*Hypera nigrirostris* (Fabricius, 1775)	2				1		3
*Hypera transsilvanica* (Petri, 1901)	3				1		4
! *Larinus centaurii* (Olivier, 1807)	1						1
! *Larinus iaceae* (Fabricius, 1775)					1		1
*Larinus obtusus* (Gyllenhal, 1835)	5	2			15	6	28
*Larinus planus* (Fabricius, 1792)						2	2
! *Larinus pollinis* (Laicharting, 1781)					1		1
*Lepyrus capucinus* (Schaller, 1783)					2		2
*Limnobaris t-album* (Linnaeus, 1758)						3	3
! *Liophloeus tessulatus* (O.F. Müller, 1776)					3		3
*Lixus bardanae* (Fabricius, 1787)	1						1
! *Lixus brevipes* (C.N.F. Brisout de Barneville, 1866)					1		1
*Lixus fasciculatus* (Boheman, 1835)						1	1
*Lixus myagri* (G.-A. Olivier, 1807)	1						1
*Lixus punctirostris* (Boheman, 1842)						1	1
! *Magdalis duplicata* (Germar, 1819)			1				1
! *Magdalis linearis* (Gyllenhal, 1827)						1	1
*Mecinus pascuorum* (Gyllenhal, 1813)		1			3		4
! *Melanobaris dalmatina* (H. Brisout, 1870)	1						1
*Miarus ajugae* (Herbst, 1795)						1	1
*Microplontus triangulum* (Boheman, 1845)					1		1
! *Mogulones geographicus* (Goeze, 1777)	1						1
*Mononychus punctumalbum* (Herbst, 1784)					3		3
*Nedyus quadrimaculatus* (Linnaeus, 1758)					1		1
*Neophytobius quadrinodosus* (Gyllenhal, 1813)	1						1
*Notaris acridulus* (Linnaeus, 1758)						1	1
! *Omiamima mollina* (Boheman, 1834)	3						3
*Omias murinus* (Boheman, 1842)					1	4	5
! *Omias puberulus* (Boheman, 1834)					1		1
! *Orchestes hortorum* (Fabricius, 1792)					1		1
*Orchestes rusci* (Herbst, 1795)			2				2
! *Otiorhynchus chrysostictus* (Gyllenhal, 1834)	3						3
! *Otiorhynchus fullo* (Schrank, 1781)	4						4
*Otiorhynchus ligustici* (Linnaeus, 1758)	9				5		14
*Otiorhynchus ovatus* (Linnaeus, 1758)	76		1		27	2	106
! *Otiorhynchus pilosus* (Gyllenhal, 1834)	5						5
! *Otiorhynchus raucus* (Fabricius, 1777)	51				9		60
*Otiorhynchus tristis* (Scopoli, 1763)	4				11		15
! *Otiorhynchus velutinus* (Germar, 1823)	9				9		18
*Phyllobius argentatus* (Linnaeus, 1758)	12		1			1	14
*Phyllobius brevis* (Gyllenhal, 1834)	1				1	2	4
! *Phyllobius contemptus* (Schoenherr, 1832)	1						1
*Phyllobius maculicornis* (Germar, 1823)		6			13	12	31
*Phyllobius pomaceus* (Gyllenhal, 1834)				2		3	5
*Phyllobius pyri* (Linnaeus, 1758)		4			47	1	52
*Phyllobius thalassinus* (Gyllenhal, 1834)						4	4
*Pissodes validirostris* (C.R. Sahlberg, 1834)					2		2
*Polydrusus cervinus* (Linnaeus, 1758)						1	1
! *Polydrusus inustus* (Germar, 1823)	3				1	5	9
! *Polydrusus mollis* (Strøm, 1768)	1			1		1	3
! *Polydrusus pilosus* (Gredler, 1866)							
*Polydrusus pterygomalis* (Boheman, 1840)					1		1
*Polydrusus tereticollis* (De Geer, 1775)	9	3	1		15		28
*Polygraphus poligraphus* (Linnaeus, 1758)			1				1
! *Pseudorchestes circumvistulanus* (Białooki, 1997)	1						1
! *Pseudorchestes ermischi* (Dieckmann, 1958)					3		3
*Pseudorchestes pratensis* (Germar, 1821)					1		1
*Rhamphus pulicarius* (Herbst, 1795)					1		1
*Rhinusa linariae* (Panzer, 1795)					1		1
*Rhyncolus ater* (Linnaeus, 1758)	1						1
*Rhinoncus leucostigma* (Marsham, 1802)	1						1
*Rhinoncus pericarpius* (Linnaeus, 1758)					1		1
*Romualdius scaber* (Linnaeus, 1758)	4				3		7
*Sciaphilus asperatus* (Bonsdorff, 1785)	6				14		20
*Scolytus intricatus* (Ratzeburg, 1837)			23				23
*Scolytus ratzeburgii* (E.W. Janson, 1856)		1					1
! *Scolytus rugulosus* (P.W.J. Müller, 1818)			1				1
*Sibinia pellucens* (Scopoli, 1772)					1	1	2
*Sibinia viscariae* (Linnaeus, 1761)					1	1	2
! *Sirocalodes quercicola* (Paykull, 1792)					1		1
! *Sitona ambiguus* (Gyllenhal, 1834)					3		3
*Sitona cylindricollis* (Fåhraeus, 1840)					2		2
*Sitona hispidulus* (Fabricius, 1777)	4			1	5		10
*Sitona humeralis* (Stephens, 1831)	2						2
*Sitona inops* (Schoenherr, 1832)	6				4	2	12
! *Sitona lateralis* (Gyllenhal, 1834)					1		1
*Sitona lineatus* (Linnaeus, 1758)		4			20		24
*Sitona longulus* (Gyllenhal, 1834)	1				1		2
*Sitona macularius* (Marsham, 1802)	4				1		5
*Sitona obsoletus* (Gmelin, 1790)					1		1
*Sitona sulcifrons* (Thunberg, 1798)	1				11		12
*Sitona suturalis* (Stephens, 1831)	3				2	2	7
! *Stephanocleonus microgrammus* (Gyllenhal, 1834)	1						1
*Strophosoma albosignatum* (Boheman, 1840)					3		3
*Strophosoma capitatum* (De Geer, 1775)	968	2	138	12	138	19	1277
*Tanymecus palliatus* (Fabricius, 1787)	1	2			3	3	9
*Tanysphyrus lemnae* (Paykull, 1792)						1	1
! *Thamnurgus caucasicus* (Reitter, 1887)					1		1
! *Trachyphloeus parallelus* (Seidlitz, 1868)	3						3
! *Trachyphloeus spinimanus* (Germar, 1823)	1						1
! *Trypodendron signatum* (Fabricius, 1792)			1				1
! *Tychius albolineatus* (Motschulsky, 1860)					1		1
! *Tychius aureolus* (Kiesenwetter, 1852)					1		1
*Tychius breviusculus* (Desbrochers des Loges, 1873)					1		1
! *Tychius flavus* (Becker, 1864)					3	1	4
*Tychius picirostris* (Fabricius, 1787)					1		1
*Tychius quinquepunctatus* (Linnaeus, 1758)		1			6		7
*Tychius sharpi* (Tournier, 1874)					1		1
! *Urometopus nemorum* (L. Arnol’di, 1969)	8						8
*Xyleborus dispar* (Fabricius, 1792)			13				13
*Zacladus geranii* (Paykull, 1800)					1		1

*—protected species from the Red Book of Russia [51], **—protected species from the Red Book of the Ryazan region [50], !—new species in the region. Note: pitfall traps (PfT), beer traps (BT), freely hanging flight intercept traps or window traps (FWT), Malaise traps (MT), pan traps (PT), and sweep net (SN).
